# Nonpathogenic *E. coli* engineered to surface display cytokines as a new platform for immunotherapy

**DOI:** 10.21203/rs.3.rs-4031911/v1

**Published:** 2024-03-22

**Authors:** Shaobo Yang, Michal Sheffer, Isabel E Kaplan, Zongqi Wang, Mubin Tarannum, Khanhlinh Dinh, Yasmin Abdulhamid, Roman Shapiro, Rebecca Porter, Robert Soiffer, Jerome Ritz, John Koreth, Yun Wei, Peiru Chen, Ke Zhang, Valeria Márquez-Pellegrin, Shanna Bonanno, Neel Joshi, Ming Guan, Mengdi Yang, Deng Li, Chiara Bellini, Jianzhu Chen, Catherine J. Wu, David Barbie, Jiahe Li, Rizwan Romee

**Affiliations:** 1 Department of Bioengineering, Northeastern University, Boston, MA; 2 Department of Medical Oncology, Dana-Farber Cancer Institute, Boston, MA; 3 Department of Chemistry and Chemical Engineering, Northeastern University, Boston, MA; 4 Department of Biomedical Engineering, University of Michigan, Ann Arbor, MI; 5 Koch Institute for Integrative Cancer Research and Department of Biology, Massachusetts Institute of Technology, Cambridge, MA

## Abstract

Given the safety, tumor tropism, and ease of genetic manipulation in non-pathogenic *Escherichia coli* (*E. coli*), we designed a novel approach to deliver biologics to overcome poor trafficking and exhaustion of immune cells in the tumor microenvironment, via the surface display of key immune-activating cytokines on the outer membrane of *E. coli* K-12 DH5α. Bacteria expressing murine decoy-resistant IL18 mutein (DR18) induced robust CD8^+^ T and NK cell-dependent immune responses leading to dramatic tumor control, extending survival, and curing a significant proportion of immune-competent mice with colorectal carcinoma and melanoma. The engineered bacteria demonstrated tumor tropism, while the abscopal and recall responses suggested epitope spreading and induction of immunologic memory. *E. coli* K-12 DH5α engineered to display human DR18 potently activated mesothelin-targeting CAR NK cells and safely enhanced their trafficking into the tumors, leading to improved control and survival in xenograft mice bearing mesothelioma tumor cells, otherwise resistant to NK cells. Gene expression analysis of the bacteria-primed CAR NK cells showed enhanced TNFα signaling via NFkB and upregulation of multiple activation markers. Our novel live bacteria-based immunotherapeutic platform safely and effectively induces potent anti-tumor responses in otherwise hard-to-treat solid tumors, motivating further evaluation of this approach in the clinic.

## Introduction

The development of recent immunotherapeutic approaches including the use of immune checkpoint blockade, chimeric antigen receptor (CAR) T cells, CAR natural killer (NK) cells, and tumor-infiltrating lymphocytes (TILs) has substantially helped to improve the treatment of patients with advanced malignancies^1–4^. However, most advanced, and metastatic malignancies remain incurable and therefore represent a major unmet need^5,6^. Lack of tumor-specific targets in most cancer types, poor tumor trafficking of the immune effector cells including CAR T and NK cells, and their dysfunction and exhaustion in the highly immune suppressive tumor microenvironment (TME) are still some of the major hurdles to further improving immunotherapeutic approaches to cancer^2,3^.

Recently, many genera of bacteria (*Escherichia*, *Salmonella*, etc.) have been reported to preferentially colonize hypoxic tumor tissues, generating renewed interest in potentially harnessing live bacteria to deliver novel payloads to modulate the TME^7^. Additionally, several early-phase clinical trials have demonstrated the feasibility and safety of using engineered bacteria, though with very limited efficacy^8–10^. Modern synthetic biology and genetic modification tools have allowed the engineering of bacteria to deliver payloads including various immune-modulating molecules^11–15^, toxins^12–14,16^, CAR T cell-stimulating tags^17^ or to guide their spatiotemporal delivery to TME^18,19^. However, most of these approaches depend on the secretion and or self-lysis of the bacteria to release the therapeutic payload which often limits their half-life and efficacy^12,15^.

Here we developed a new immunotherapy platform for the preferential TME delivery and enhanced exposure and retention of key immune-activating cytokines by assembling them on the outer membrane of tumor tropic and non-pathogenic bacteria *E. coli* K-12 DH5α. While bacterial surface display technologies have found wide applications in antibody-antigen screening and bio-catalysis^20,21^, the use of this technology to display immune-activating cytokines for cancer immunotherapy has not been previously explored. We hypothesized that the expression of these molecules on the bacterial outer membrane would enhance their ability to effectively induce potent immune responses through surface anchoring and clustering, while the use of non-pathogenic facultative anaerobe bacteria would mediate their safe, efficient, and preferential delivery into the TME. The *E. coli* K-12 DH5α, originally isolated from native gut microbiota in humans, is non-pathogenic with no major toxins reported (BSL-1 strain)^22^. Furthermore, these bacteria are sensitive to the commonly used antibiotics (pan-sensitive) thus providing an additional layer of safety for their potential clinical applications^23^.

We successfully expressed murine and human IL15, IL18, decoy-resistant IL18 mutein (DR18), and IL21 on the outer membrane of *E. coli* K-12 DH5α with multiple bacterial scaffolds. The bacteria displaying DR18 induced the most potent CD8^+^ T and NK cell-dependent immune responses in tumor-bearing immune-competent syngeneic mouse models and in combination with human CAR NK cells in tumor-bearing xenograft mice demonstrating their ability to enhance CAR NK cell tumor trafficking and thus further broadening the translational potential of this approach. Moreover, gene expression analysis provided clues to the underlying mechanisms mediating their CAR NK cell activation. In summary, we here describe the development of a novel bacterial therapy platform by successfully engineering non-pathogenic *E. coli* K-12 DH5a to display potent cytokines for enhanced immunotherapy.

## Results

### *E. coli* engineered to display cytokines

We hypothesized that engineering non-pathogenic facultative anaerobe *E. coli* K-12 DH5α would allow us to display highly activating cytokines on its outer membrane and thereby deliver them preferentially to the TME and trigger a potent immune response. IL12, IL15, IL18 (and its muteins), and IL21 are some of the most potent immune-activating cytokines, demonstrating promising activity alone and or in combination with other immunotherapeutic approaches in pre-clinical tumor models and patients with advanced malignances^24^. Additionally, IL18 muteins, such as the decoy-resistant IL18 (DR18), were developed for decreased binding affinity to the IL18 binding protein thus promoting its interaction with the IL18 receptor^25^. Despite the promising efficacy of these cytokines, short half-life, lower effective concentrations in the TME and systemic toxicity are some of the major limitations hampering their therapeutic application^24^.

**Extended Data Fig. 1 |:**
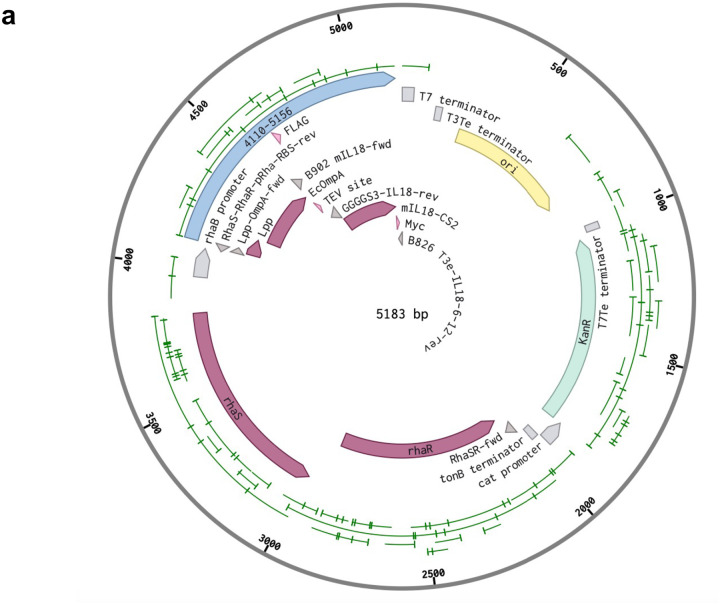
**a**, Representative plasmid map includes OmpA (EcOmpA) scaffold and mDR18 (mIL18-CS2).

**Fig. 1 |:**
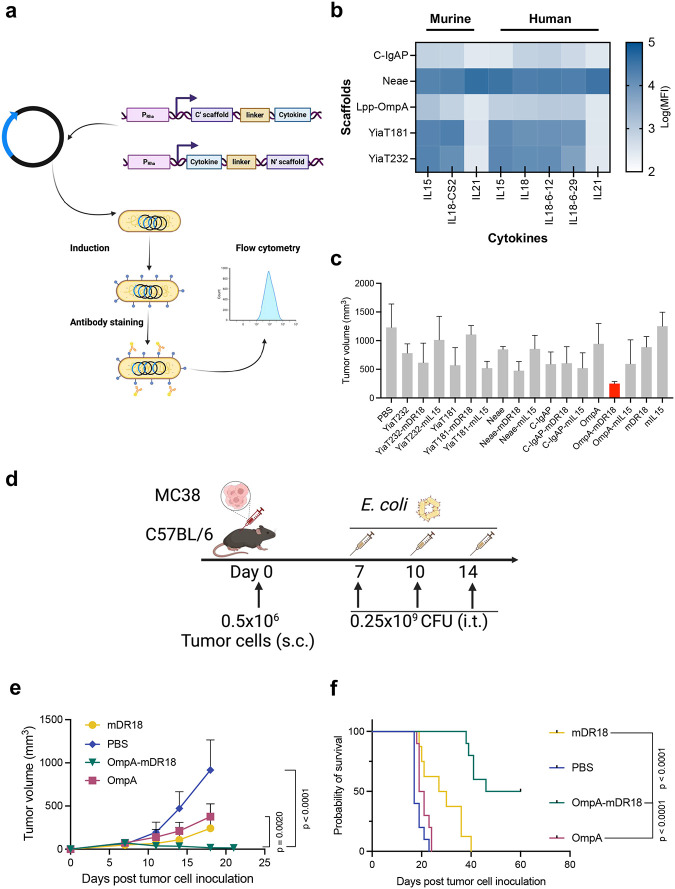
Murine decoy-resistant IL18 displayed by bacterial scaffold Lpp-OmpA induces potent anti-tumor responses in an immune-competent syngeneic mouse model (MC38). **a**, Schematic figure showing the surface display of cytokines in *E. coli* by their transformation with the cytokine-scaffold coding plasmids and the surface expression assessed by flow cytometry. **b**, Heatmap showing the expression levels of 8 cytokines (murine interleukin-15, mIL15; decoy resistant murine interleukin-18, mIL18-CS2; murine interleukin-21, mIL21; human IL15, hIL15; human IL18, hIL18; two types of human decoy resistant IL18, human hIL18–6-12 and, hIL18–6-29; human IL21, hIL21) displayed by 5 different bacterial scaffolds: C-IgAP, Neae, Lpp-OmpA (OmpA), YiaT181 and, YiaT232. The display levels represent median fluorescence intensity (MFI) measured by flow cytometry. **c**, Mean tumor volumes as assessed on day 15 post-tumor inoculation in different groups of mice treated with PBS, mIL18-CS2 (mDR18, 0.4mg/kg), mIL15 (0.4mg/kg), bacteria displaying mDR18, mIL15 or scaffold only by C-IgAP, Lpp-OmpA, YiaT181, YiaT232 or Neae (10^9^ CFU) intratumorally (i.t.) three times on days 7, 10 and 14 (n = 6 each group)]. **d**, C57BL/6 mice were subcutaneously (s.c.) engrafted with 0.5 × 10^6^ MC38 cells on one side of the flank. Starting on day 7 after tumor cell injection (tumor size 50–100 mm^3^), mice were treated with PBS, mDR18 (mIL18-CS2, 4mg/kg), OmpA (0.25 × 10^9^ CFU), OmpA-mDR18 (0.25 × 10^9^ CFU). **e**, **f**, Mean tumor growth (**e**) and, Kaplan–Meier survival curves (**f**) for mice (n = 8–10 each group) bearing MC38 tumors after treatment with OmpA-mDR18 (0.25 × 10^9^ CFU), OmpA (0.25 × 10^9^ CFU), mDR18 (4mg/kg) and PBS. Two-way ANOVA test for tumor growth curve (**e**) and Mantel-Cox test for survival curve (**f**). Data represent means ± SEM (**c**, **e**).

To overcome these major hurdles, we engineered *E. coli* K-12 DH5α to surface display these major cytokines. For this we chose five widely used bacterial scaffolds: Lipoprotein fused with *E. coli* Outer membrane protein A (Lpp-OmpA), C terminal of IgA proteinase (C-IgAP), N terminal of eaeA (Neae), YiaT protein with the N terminal 1–232 amino acids (YiaT232) and YiaT protein with the N terminal 1–181 amino acids (YiaT181)^26–28^. We engineered these scaffolds to present 8 different cytokines: murine interleukin-15 (mIL15), murine decoy resistant interleukin-18-CS2 (mDR18), murine interleukin-21 (mIL21), human interleukin-15 (hIL15), human interleukin-18 (hIL18), human decoy resistant interleukin-18–6-12 (hIL18–6-12), human decoy resistant interleukin-18–6-29 (hIL18–6-29) and human interleukin-21 (hIL21). We cloned these cytokine-scaffold fusion constructs into pLygo, pDS861, or pDSG232 plasmids using rhamnose or tetracycline-inducible promoters (P_rha_ or P_tet_). *E. coli* K-12 DH5α was then transformed with these plasmids and the expression of the cytokines was assessed by flow cytometry after staining the bacteria with the corresponding antibodies (Fig. 1a, Extended Data Fig. 1). Human and murine IL15, IL18, and decoy-resistant IL18 were all expressed regardless of the scaffold used, while human and murine IL21 could only be displayed by Neae (Fig. 1b). We were unable to display IL12 (data not shown), most likely due to its post-translation modifications requiring a eukaryotic cell system. Based on these results, we decided to focus on bacteria displaying murine and human IL15, IL18, and DR18 for further evaluation.

**Extended Data Fig. 2 |:**
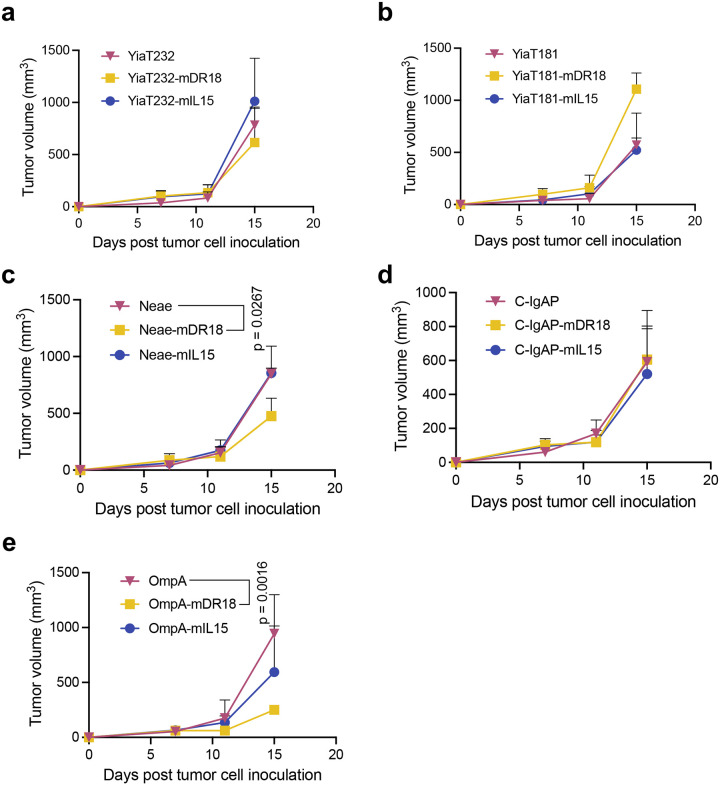
Murine decoy-resistant IL18 displayed by bacteria inhibit tumor growth in an immune-competent syngeneic mouse model (MC38). **a**-**e**, Mean tumor growth in mice bearing MC38 tumors after being treated with YiaT232, YiaT181, OmpA, Neae and C-IgAP displaying nothing, mDR18 or mIL15. Data are representative of one biological independent experiment, with n = 6 tumors per group. Two-way ANOVA test. Data represent means ± SEM (**a**-**e**).

To compare their ability to induce an immune response, we first screened these leading candidates (murine IL15, IL18, and DR18 with different bacterial scaffolds) in an immune-competent mouse model, C57BL/6 bearing the syngeneic colon cancer cell line MC38 (murine colorectal carcinoma cell line). 0.5 million MC38 cells were implanted into both the flanks (subcutaneous) of each mouse and starting from day 7 when the tumor volume reached 50–100 mm^3^, one billion colony-forming units (CFU) of bacteria were injected intratumorally (i.t.) into each tumor on days 7, 10, and 14. The bacteria with murine decoy-resistant IL18 displayed by OmpA (OmpA-mDR18) demonstrated the most effective tumor control and were therefore chosen for further assessment (Fig. 1c, Extended Data Fig. 2a–e).

**Extended Data Fig. 3 |:**
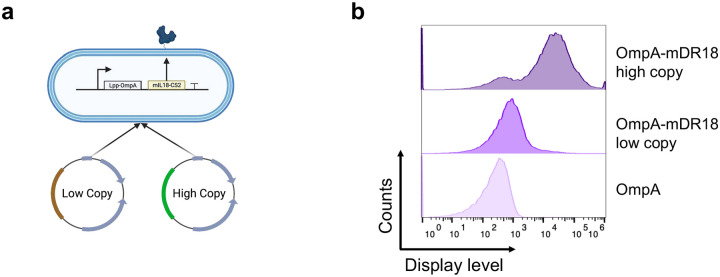
The display levels of mDR18 are significantly increased by switching the replication origin in the plasmid. **a**, The schematic figure shows an increase in the surface display levels of mIL18-CS2 (mDR18) by increasing the copy number of plasmids. **b**, Representative flow cytometry histograms of mDR18 levels on the surface of bacteria.

Before further evaluation of the OmpA-mDR18 system, we increased its expression levels by optimizing the induction conditions and switching to a high-copy plasmid backbone with an alternate replication origin (Extended Data Fig. 3). All subsequent experiments were performed using these optimized OmpA-mDR18 bacteria. To further evaluate the ability of OmpA-mDR18 to control both the tumor growth and improve survival in these animals, we treated C57BL/6 mice bearing subcutaneous (unilaterally in flanks) MC38 with the following treatment groups: OmpA-mDR18 bacteria, OmpA (bacteria with empty scaffold), mDR18 (purified murine DR18), and PBS, all injected intratumorally on days 7, 10 and 14 (Fig. 1d). The optimized OmpA-mDR18 bacteria in these mice demonstrated very effective anti-tumor responses even with a lower bacterial dose (0.25 billion versus 1 billion CFU of the non-optimized bacteria tested previously, Fig. 1c). In these mice, tumors were non-palpable by ~day 21 post-tumor inoculation in most mice treated with OmpA-mDR18, with 50% of these mice remaining tumor-free beyond day 100. OmpA-mDR18 bacteria were more potent than the purified mDR18 in these mice and demonstrated highly effective tumor control, with a median survival of 53 days versus 28.5 days in mice receiving purified mDR18 (p < 0.0001) (Fig. 1e–f). Furthermore, OmpA-mDR18 bacteria at 0.25 billion CFU dose used in these experiments were safe. At a higher dose (1 billion CFU), however, even though tumor control was more dramatic, most (~80%) of the mice demonstrated weight loss, hunched backs, or slowed movements within days after the bacterial injections, potentially indicating induction of a hyper-inflammatory response at these doses.

**Extended Data Fig. 4 |:**
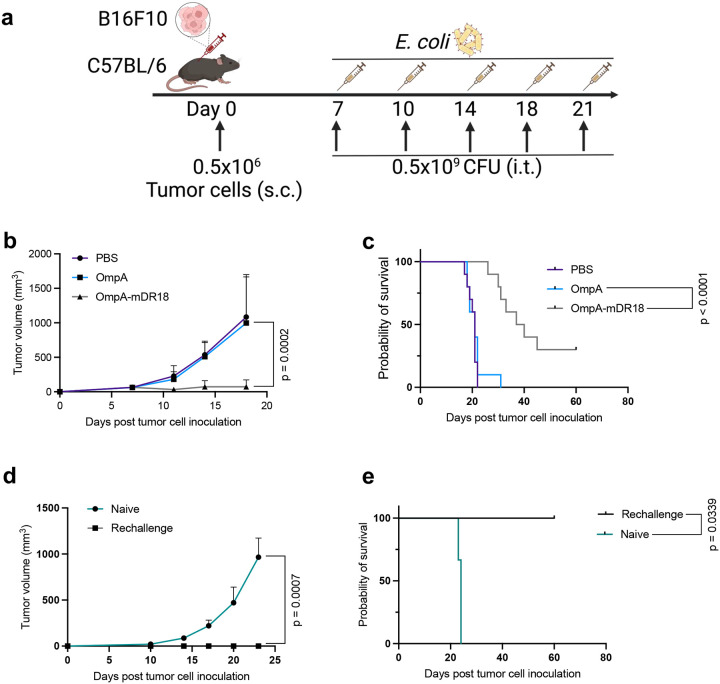
OmpA-mDR18 inhibits tumor growth in an immune-competent syngeneic melanoma mouse model (B16F10). **a**, C57BL/6 mice were subcutaneously (s.c.) engrafted with 0.5 × 10^6^ B16F10 cells, starting on day 7 (tumor size reaches 40 – 70 mm^3^), mice were treated with PBS (n = 10), OmpA (0.5 × 10^9^ CFU, n = 10) or OmpA-mDR18 (0.5 × 10^9^ CFU, n = 10) (i.t.) five times (day 7, day 11 and day 14, day 18 and day 21). **b**, **c**, Mean tumor growth (**b**) and Kaplan–Meier survival curves (**c**) for mice bearing B16F10 tumors after treatment. **d**, **e**, Mean tumor growth (**d**) and Kaplan–Meier survival curves (**e**) of cured C57BL/6 mice (n = 3) obtained from mice treated with OmpA-mDR18 and naïve C57BL/6 (gender and age-matched with cured mice) were subcutaneously (s.c.) engrafted with 0.5 × 10^6^ B16F10 cells on the other side of flanks. Two-way ANOVA test for tumor growth curve (**b**, **d**) and Mantel-Cox test for survival curve (**c**, **e**). Data are representative of two independent experiments, with n = 10 mice per group (**b**, **c**). Data represent means ± SEM (**b**, **d**).

To assess the broader applicability of the OmpA-mDR18 bacteria, we also evaluated them in a melanoma tumor model with C57BL/6 mice bearing B16F10 tumor cells. Since B16F10 is widely resistant to immunotherapy due to the downregulation of MHC-I and lack of MHC-II expression^29^, we used five (two times weekly) injections in this model, with 0.5 billion CFU per dose (Extended Data Fig. 4a). Again, we observed substantial tumor control and improved survival in the OmpA-mDR18 bacteria treatment group, with a median survival of 38 days in the OmpA-mDR18 versus 21 days in the OmpA group (p < 0.0001). ~30% of mice in the OmpA-mDR18 group stayed tumor-free until 60 days (last follow-up) and thus were deemed “cured” (Extended Data Fig. 4b, c).

**Extended Data Fig. 5 |:**
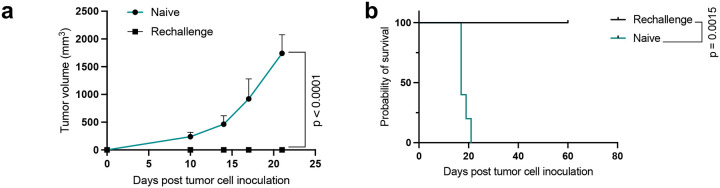
Treatment with the engineered bacteria induces immunological memory leading to enhanced recall responses. **a**, **b**, C57BL/6 mice (n = 5) cured from MC38 upon treatment with OmpA-mDR18 and naïve C57BL/6 (gender and age-matched with the cured mice) subcutaneously (s.c.) engrafted with 0.5 × 10^6^ MC38 cells and then monitored for tumor growth and survival. Mean tumor growth (**a**) and Kaplan– Meier survival curves (**b**) for mice injected with 0.5 × 10^6^ MC38 cells on Day 0. Two-way ANOVA test for tumor growth curve (**a**) and Mantel-Cox test for survival curve (**b**). Data are representative of one independent experiment, with n = 5 mice per group (**a**, **b**). Data represent means ± SEM (**a**).

To address if the *in vivo* anti-tumor responses induced by the OmpA-mDR18 bacteria were associated with the induction of immunologic memory to the tumors, we rechallenged tumor-free animals from the above experiment with MC38 on the contralateral flank two months after the initial clearance of tumors. All mice in the rechallenged group remained tumor-free for at least 60 days after their tumor inoculation, thus indicating the induction of a strong immunologic memory to these tumors (Extended Data Fig. 5a, b). We also evaluated recall responses in the B16F10 model in a similar manner and as before, all rechallenged mice remained tumor-free by the study endpoint (60 days after rechallenging: Extended Data Fig. 4d, e).

**Fig. 2 |:**
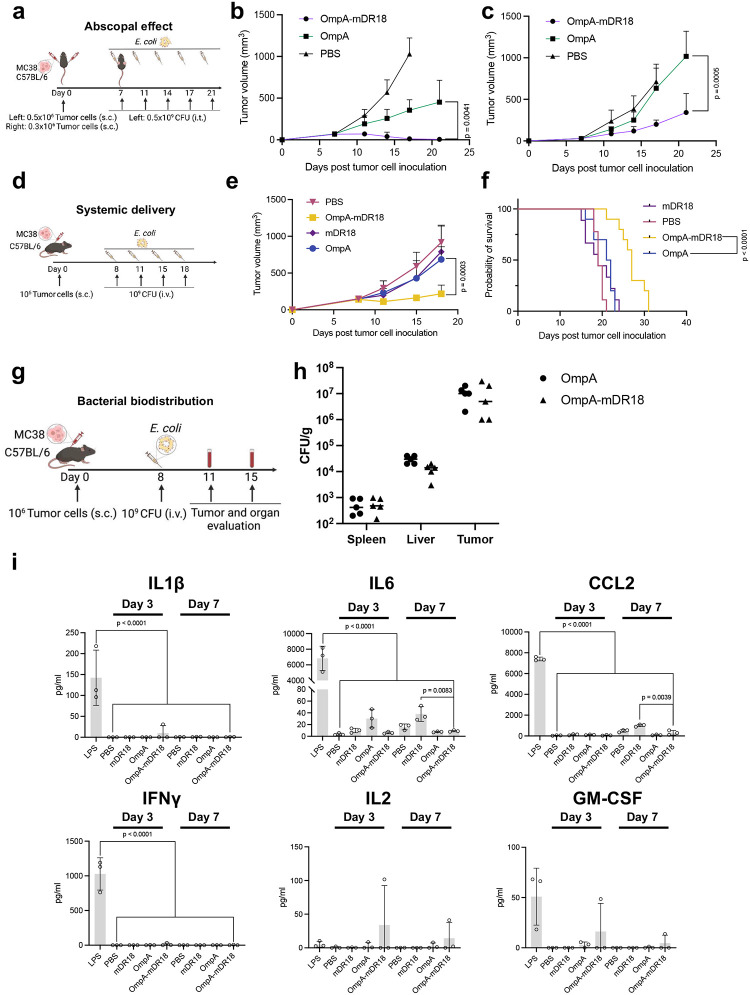
Systemically delivered tumor-homing OmpA-mDR18-*E. coli* induce an abscopal effect and are safe. **a**, Mice (n = 5 each group) were subcutaneously (s.c.) engrafted with 0.5 × 10^6^ and 0.3 × 10^6^ MC38 cells into the left and right flanks. Starting from day 7, tumors on the left side were treated with PBS, OmpA-mDR18 (0.5 × 10^9^ CFU), or OmpA (0.5 × 10^9^ CFU) (i.t.) five times on days 7, 11, 14, 17, and 21. **b**, **c**, Mean tumor growth on the treated side (**b**), and untreated side (**c**) of mice. **d**, Mice (n = 8–10 each group) engrafted with 10^6^ MC38 cells were treated on days 8, 11, 15 and 18 intravenously (i.v.). **e**, **f**, Mean tumor growth (**e**) and Kaplan–Meier survival curves (**f**) of mice. **g**, On day 8 after engraftment of 10^6^ MC38 cells, mice (n = 3–5 each group) were treated once (i.v.). Plasma was isolated from blood collected from the submandibular vein on days 11 and 15 for cytokine measurement. Tissues were collected on day 15 for bio-distribution. **h**, Bacterial distribution in tumor, liver, and spleen on 7 days post-injection. CFU/g is bacterial concentration. **i**, The concentration of cytokines associated with CRS: interleukin-1β (IL1β), interleukin-6 (IL6), monocyte chemoattractant protein-1 (CCL2), interferon-γ (IFNγ), interleukin-2 (IL2) and granulocyte macrophage colony-stimulating factor (GM-CSF) in the plasma (on days 3 and 7 post-treatment unless other mentioned) from mice treated with LPS (5-hour post-treatment), PBS, mDR18, OmpA and OmpA-mDR18. Mice were treated with PBS, 4mg/kg mDR18, 10^9^ CFU OmpA or OmpA-mDR18 (**d**, **e**, **f**, **g**, **h**). Two-way ANOVA test for growth curve (**b**, **c**, **e**), Mantel-Cox test for survival curve (**f**), one-way ANOVA test and unpaired t-test for comparison (**i**). Data represent means ± SEM (**b**, **c**, **e**, **i**).

The strong recall responses seen in these immune-competent models implied the induction of an adaptive immune response and possibly epitope spreading typically mediated by T cells. To investigate this possibility, we evaluated abscopal responses induced by the engineered bacteria by first injecting MC38 tumor cells (subcutaneously, 0.5 million on the left and, 0.3 million cells on the right flank) of each animal. When the tumor volume of the right flank reached 50–100 mm^3^, 0.5 billion CFU bacteria were injected only into the left flank tumors on days 7, 11, 14, 17, and 21 and the tumor volumes were assessed (Fig. 2a). The mice treated with OmpA-mDR18 bacteria had improved tumor control when compared to mice treated with non-mDR18 bacteria (OmpA) on both the treated (p = 0.0041, left flank) and un-treated (p = 0.0005, right flank) sides supporting the induction of an abscopal effect and thus a strong systemic immune response (Fig. 2b, c).

In summary, we successfully displayed key activating cytokines on the outer membrane of *E. coli,* with mDR18 displayed by the OmpA scaffold (OmpA-mDR18) inducing very potent tumor control (and prolonging survival), recall, and systemic responses in two immune-competent mouse models. Furthermore, the induction of immunological memory and abscopal effects underscores the high translation potential of this novel immunotherapeutic system.

### Tumor enrichment, efficacy, and safety

**Extended Data Fig. 6 |:**
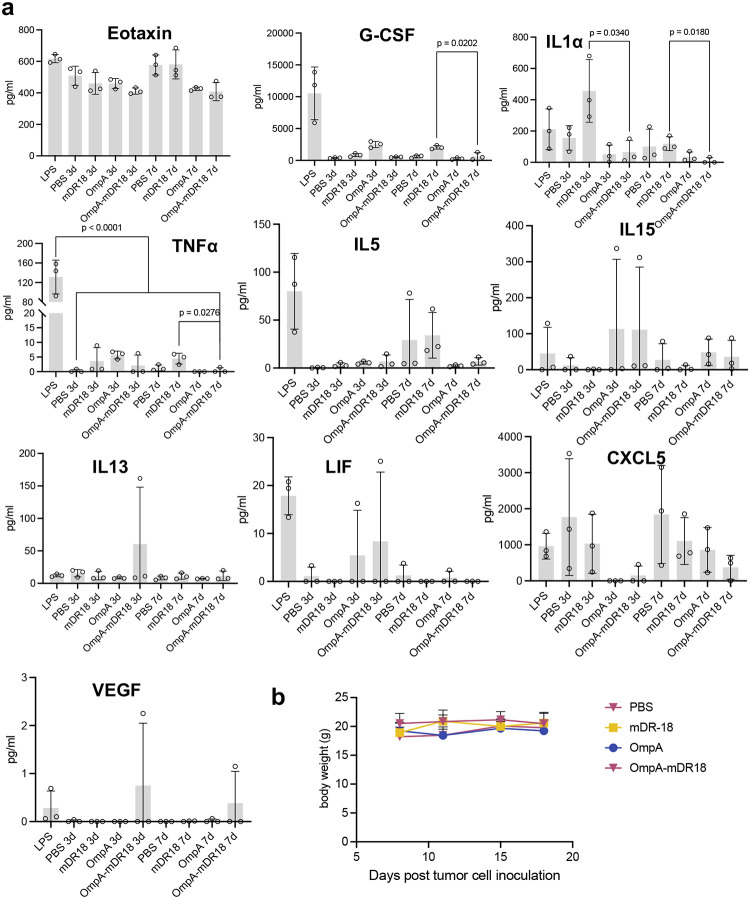
The concentration of key cytokines and chemokines and body weight in immune-competent mice upon treatment with OmpA-mDR18 and other controls. **a**, The concentration of Eotaxin, granulocyte colony stimulating factor (G-CSF), interleukin-1α (IL1α), tumor necrosis factor alpha (TNF-α), interleukin-5 (IL5), interleukin-13 (IL13), leukemia inhibitory factor (LIF), C-X-C motif chemokine ligand 5 (CXCL5) and vascular endothelial growth factor (VEGF) in the plasma isolated from blood collected from submandibular vein. *p < 0.05; **p < 0.01; ****p < 0.0001, one-way ANOVA test for multi-comparison and unpaired t-test for single comparison (**a**). **b**, Body weight of mice mentioned in Fig. 3d. Data represent means ± SEM (**a**, **b**).

To assess the feasibility, safety, and potential efficacy of systemically delivered engineered bacteria, we used C57BL/6 mice bearing MC38 tumors as above but infused the bacteria through tail vein injections (Fig. 2d). The mice subsequently received intravenous (i.v.) injections of OmpA-mDR18 expressing *E. coli* (1 × 10^9^ CFU), or controls (Fig. 2d) on days 8, 11, 15, and 18. The mice received treatment after their tumor volume was larger than 100mm^3^ (typically by day 8) as a previous study showed systemically delivered E. *coli* efficiently home into the tumors of this volume in mice^30^. We saw better tumor control and survival benefit in mice that received OmpA-mDR18 versus OmpA-expressing bacteria (p = 0.0003) and neither OmpA nor mDR18 led to any tumor control on their own (Fig. 2e, f). Furthermore, despite systemic delivery of the live bacteria, none of the mice treated with bacteria in our study developed any obvious toxicity as assessed by weight loss, hunched posture, or unexpected death (Extended Data Fig. 6b). 20% of mice (2 of 10) treated with the purified recombinant cytokine (mDR18) were found to have liver failure and hydroperitoneum upon euthanasia, suggesting considerable toxicity upon systemic delivery of this cytokine.

**Extended Data Fig. 7 |:**
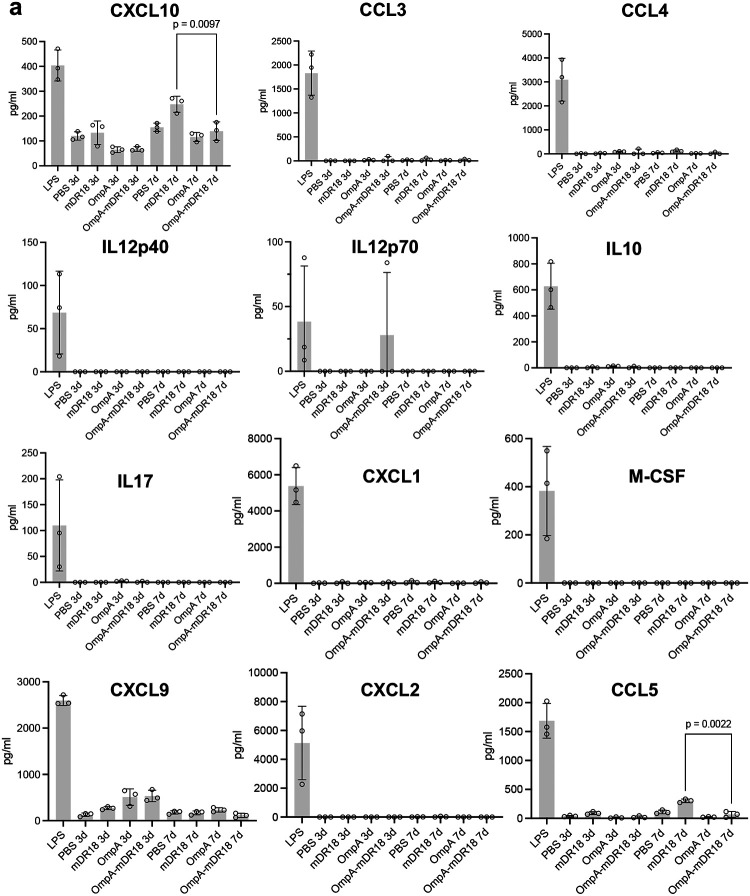
The concentration of key cytokines and chemokines in immune-competent mice upon treatment with OmpA-mDR18 and other controls. **a**, The concentration of C-X-C Motif Chemokine Ligand 10 (CXCL10), Chemokine ligand 3 (CCL3), Chemokine ligand 4 (CCL4), interleukin-12 p40 (IL12p40), interleukin-12 p70 (IL12p70), interleukin-10 (IL10), interleukin-17 (IL17), C-X-C Motif Chemokine Ligand 1 (CXCL1), Macrophage colony-stimulating factor (M-CSF), C-X-C Motif Chemokine Ligand 9 (CXCL9), C-X-C Motif Chemokine Ligand 2 (CXCL2), and Chemokine ligand 5 (CCL5) in the plasma isolated from blood collected from submandibular vein. Unpaired t-test. Data represent means ± SEM (**a**).

Because of the lack of side effects and potent tumor control with the systemic delivery of the engineered bacteria, we hypothesized that these bacteria were preferentially enriched in the TME due to their facultative anaerobic nature. To test this hypothesis, we injected OmpA-mDR18 bacteria intravenously through the tail vein in another batch of C57BL/6 mice bearing MC38 tumors. Upon sacrifice on day 15, various organs and tumors were harvested for the enumeration of CFU (Fig. 2g). In agreement with our hypothesis, we observed preferential enrichment of engineered bacteria in the tumors of the treated mice (Fig. 2h). The lack of any toxicity was also consistent with the cytokine profiles from the treated mice (purified LPS treated mice as positive control) showing no major increase in the cytokines typically associated with the development of cytokine release syndrome (CRS) in the mice after i.v. injection of the OmpA-mDR18 displaying *E. coli*^31,32^ (Fig. 2g, i, Extended Data Fig. 6a, and Extended Data Fig. 7). However, we observed increased levels of some inflammation-relevant proteins in the mice treated with the purified cytokine (mDR18) but not with engineered bacteria, consistent with systemic toxicity seen in mice treated with this cytokine (Fig. 2i, Extended Data Fig. 6a and Extended Data Fig. 7). The results from these experiments highlight the potential safety of the engineered bacteria even when delivered systemically in future clinical trials.

### Dependence on NK cells and CD8^+^ T cells

**Extended Data Fig. 8 |:**
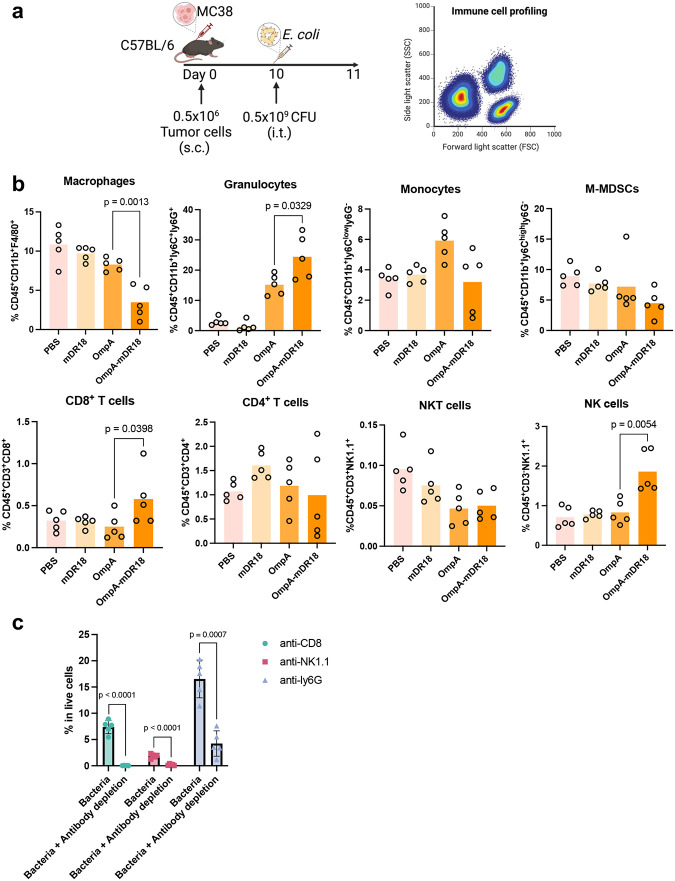
OmpA-mDR18 rapidly promote tumor-infiltrating NK and CD8^+^ T cells in MC38 model. **a**, C57BL/6 mice were subcutaneously (s.c.) engrafted with 0.5 × 10^6^ MC38 cells. When tumor size reached 150 – 200 mm^3^ on day 10, mice were treated with PBS (n = 5), OmpA-mDR18 (0.5 × 10^9^ CFU, n = 5), OmpA (0.5 × 10^9^ CFU, n = 5) or mDR18 (4mg/kg, n = 5) intratumorally (i.t.). Tumor tissues were harvested for further analysis for tumor-infiltrating immune cells (CD8^+^ T cells, CD4^+^ T cells, NK cells, NKT cells, macrophages, monocytes, granulocytes, and mononuclear myeloid-derived suppressor cells, M-MDSCs) 1 day after treatment by flow cytometer. **b**, The percentage of key cell types (as a proportion of live cells): CD8^+^ T cells (CD45^+^CD3^+^CD8^+^), CD4^+^ T cells (CD45^+^CD3^+^CD4^+^), NK cells (CD45^+^CD3^−^NK1.1^+^), T like NK cells (NKT, CD45^+^CD3^+^NK1.1^+^), granulocytes (CD45^+^CD11b^+^Ly6C^+^ Ly6G^+^), monocytes (CD45^+^CD11b^+^Ly6C^low^Ly6G^−^), macrophages (CD45^+^CD11b^+^F4/80^+^) and M-MDSCs (CD45^+^CD11b^+^Ly6C^high^Ly6G^−^) in tumor microenvironment were shown in the bar graph. Unpaired t-test. **c**, Summary of the data showing the percentage of CD8^+^ T cells, NK cells, and granulocytes as a proportion of CD45^+^ cells blood cells in mice bearing MC38 on day 1 after being treated with bacteria (i.t.) or bacteria (i.t.) plus monoclonal antibody (i.p.).

**Fig. 3 |:**
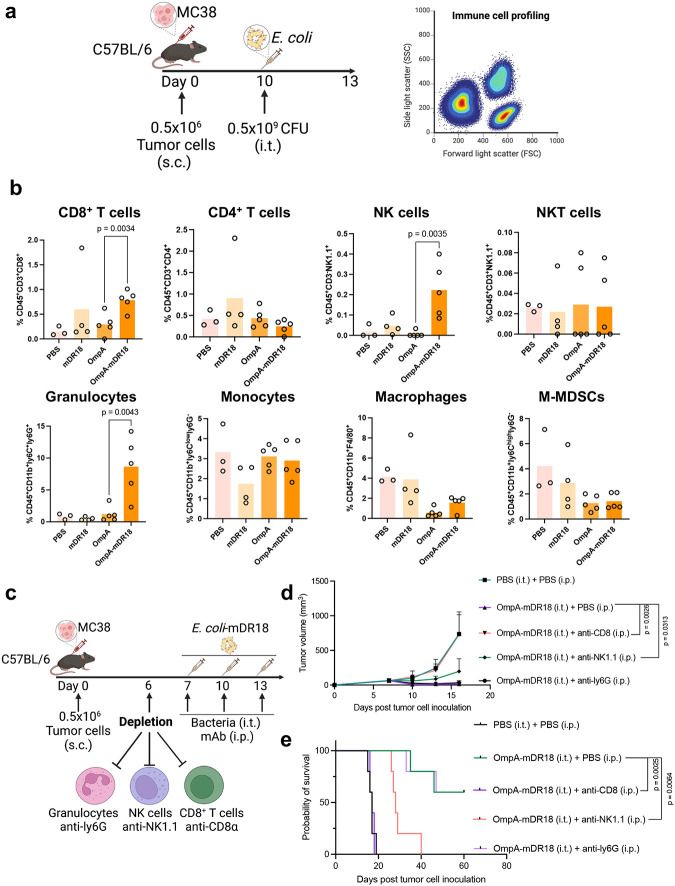
Tumor control by OmpA-mDR18 is mediated by CD8^+^ T cells and Natural Killer (NK) cells in the tumor microenvironment (TME). **a**, C57BL/6 mice were subcutaneously (s.c.) engrafted with 0.5 × 10^6^ MC38 cells in the flanks. When tumor size reached 150–200 mm^3^ on day 10, mice (n = 3–5 each group) were treated with PBS, OmpA-mDR18 (0.5 × 10^9^ CFU), OmpA (0.5 × 10^9^ CFU) or mDR18 (4mg/kg) intratumorally (i.t.). The mice were sacrificed, and the tumors harvested for flow cytometric analysis of tumor-infiltrating immune cells (CD8^+^ T cells, CD4^+^ T cells, NK cells, NKT cells, macrophages, monocytes, granulocytes, and mononuclear myeloid-derived suppressor cells, M-MDSCs) 3 days after the bacterial injection. **b**, The percentages of different cell types (as a proportion of live cells): CD8^+^ T cells (CD45^+^CD3^+^CD8^+^), CD4^+^ T cells (CD45^+^CD3^+^CD4^+^), NK cells (CD45^+^CD3^−^NK1.1^+^), T like NK cells (NKT, CD45^+^CD3^+^NK1.1^+^), granulocytes (CD45^+^CD11b^+^Ly6C^+^Ly6G^+^), monocytes (CD45^+^CD11b^+^Ly6C^low^Ly6G^−^), macrophages (CD45^+^CD11b^+^F4/80^+^) and M-MDSCs (CD45^+^CD11b^+^Ly6C^high^Ly6G^−^) in the tumor microenvironment. **c**, C57BL/6 mice (n = 5 each group) were subcutaneously (s.c.) engrafted with 0.5 × 10^6^ MC38 cells and on day 6, treated with monoclonal antibodies anti-CD8α, anti-NK1.1, anti-ly6G, or PBS intraperitoneally (i.p.) and then treated with OmpA-DR18 (0.5 × 10^9^ CFU) or PBS intratumorally (i.t.) on days 7, 10, and 13. **d**, **e**, Mean tumor growth (**d**) and Kaplan–Meier survival curves (**e**) for mice bearing MC38 treated with monoclonal antibodies or PBS, and bacteria or PBS. Unpaired t-test for percentage data (**b**), two-way ANOVA test for tumor growth curve (**d**) and Mantel-Cox test (**e**) for survival curve. Data represent means ± SEM (**b**, **d**).

The potent anti-tumor responses along with the recall and abscopal effects induced with the engineered bacteria above in immune-competent animals suggested activation of the adaptive immune system *in vivo*. To investigate the potential impact of bacterial therapy on immune cells in the TME, we first performed a detailed profiling of immune cells within the tumors after bacterial injections. Specifically, on days 1 and 3 after a single intra-tumoral injection (OmpA, OmpA-mDR18, mDR18, or PBS, in two separate cohorts of mice), the subcutaneous MC38 tumors were harvested after euthanasia, and single cells were stained with a broad antibody panel for flow cytometry analysis (day 1 and day 3 post-treatment: Fig. 3a and Extended Data Fig. 8a). We observed an increase in the CD8^+^ T cells (p = 0.0034), NK cells (p = 0.0035), and granulocytes (p = 0.0043) infiltrating the tumors after treatment with the OmpA-mDR18 bacteria versus controls (OmpA bacteria) (Fig. 3b). We also observed a decrease in the number of Tumor-Associated Macrophages (TAMs, p = 0.0024, PBS versus OmpA-mDR18) and M-MDSCs (p = 0.0479, PBS versus OmpA-mDR18) in the mice treated with OmpA-mDR18 (and OmpA to a lesser extent) bacteria, potentially indicating a broader TME modulation beyond the effects on T and NK cells (Fig. 3b and Extended Data Fig. 8b).

**Extended Data Fig. 9 |:**
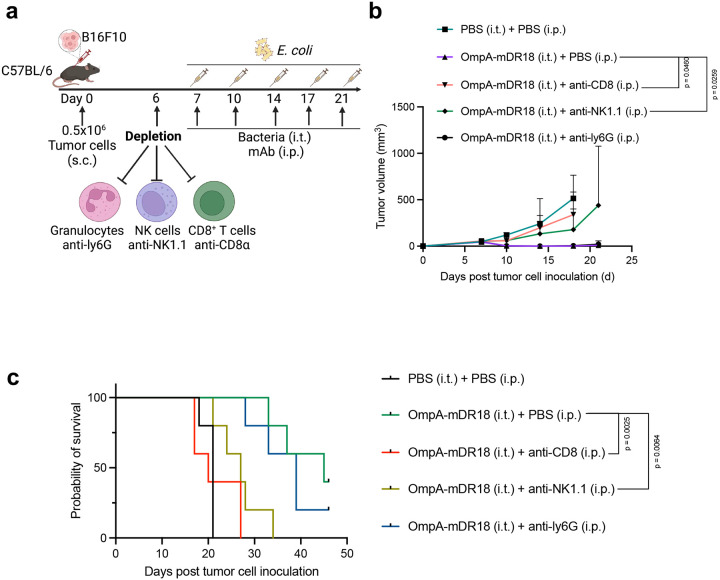
Tumor control by OmpA-mDR18 in B16F10 is mediated by CD8^+^ T cells and Natural Killer (NK) cells in the tumor microenvironment (TME). **a**, C57BL/6 mice were subcutaneously (s.c.) engrafted with 0.5 × 10^6^ B16F10 cells. On day 6, mice were treated with monoclonal antibodies anti-CD8α, anti-NK1.1, anti-ly6G, and PBS intraperitoneally (i.p.). When tumor size reached 40 – 70 mm^3^ on day 7, mice were treated with OmpA-DR18 (0.5 × 10^9^ CFU) or PBS intratumorally (i.t.) and monoclonal antibody (mAb) intraperitoneally (i.p.) on day 7, day 10, day 14, day 17 and day 21. **b, c**, Mean tumor growth (**b**) and Kaplan–Meier survival curves (**c**) for mice bearing B16F10 treated with monoclonal antibodies or PBS, and bacteria or PBS. Two-way ANOVA test for tumor growth curve (**b**) and Mantel-Cox test for survival curve (**c**). Data are representative of one independent experiment, with n = 5 mice per group. Data represent means ± SEM (**b**).

To confirm the potential contribution of T cells, NK cells, and granulocytes to the anti-tumor responses induced by OmpA-mDR18 engineered bacteria, we depleted the relevant cell populations using anti-NK1.1 (NK cell depletion), CD8a (CD8^+^ T cell depletion), and anti-ly6G (granulocytes depletion) monoclonal antibodies, respectively (Fig. 3c). Briefly, the MC38 tumor cells were implanted into the flanks of C57BL/6 mice, and then divided into the groups receiving PBS (control) or different depletion monoclonal antibodies (mAb) intraperitoneally (i.p.) on days 6, 7, 10, and 13. Meanwhile, 0.5 × 10^9^ CFU of OmpA-mDR18 bacteria were administered intratumorally (i.t.) on days 7, 10, and 13 post-tumor cell inoculation. In parallel, a separate group of mice was treated with PBS intraperitoneally and intratumorally with a similar schedule as mAbs and engineered bacteria as a negative control. The depletion of the specific cell types was confirmed by the peripheral blood peripheral blood mononuclear cells (PBMCs) collected from mice in different treatment groups on day 14 and evaluated by flow cytometry (Extended Data Fig. 8c). Consistent with the data above, we detected potent tumor control with ~60% of the mice deemed cured and still alive (as of the last follow-up) in the group treated with OmpA-mDR18 (i.t.) and PBS (i.p.). We observed no impact of granulocyte depletion (anti-ly6G), whereas the depletion of NK cells (p = 0.0064, median survival = 28) and CD8^+^ T cells (p = 0.0025, median survival = 17) led to a major decrease in tumor control, thereby underscoring the importance of these two cell types towards mediating the anti-tumor responses seen with our engineered bacteria in these mice (Fig. 3d). Compared to the negative control group (PBS, i.t. and PBS, i.p.), the depletion of CD8^+^ T cells (anti-CD8a) resulted in almost complete abrogation while as depletion of NK cells (anti-NK1.1) resulted in only partial loss of tumor control in these mice (Fig. 3d, e). We also performed similar experiments in the B16F10 model using similar mAbs and bacterial doses and schedules (Extended Data Fig. 9a). Again, we saw minimal to no impact of the granulocyte depletion while the depletion of NK cells (p = 0.0064, median survival = 27) and CD8^+^ T (p = 0.0025, median survival = 20) cells both eliminated the treatment efficacy (Extended Data Fig. 9b, c).

The data from these mechanistic studies potentially point towards OmpA-mDR18 *E. coli* mediating significant TME modulation and impacting multiple immune cells, with CD8^+^ T cells and NK cells being the major effector cells responsible for inducing potent anti-tumor responses by these engineered bacteria.

### Human DR18 bacteria and CAR NK cells

**Extended Data Fig. 10 |:**
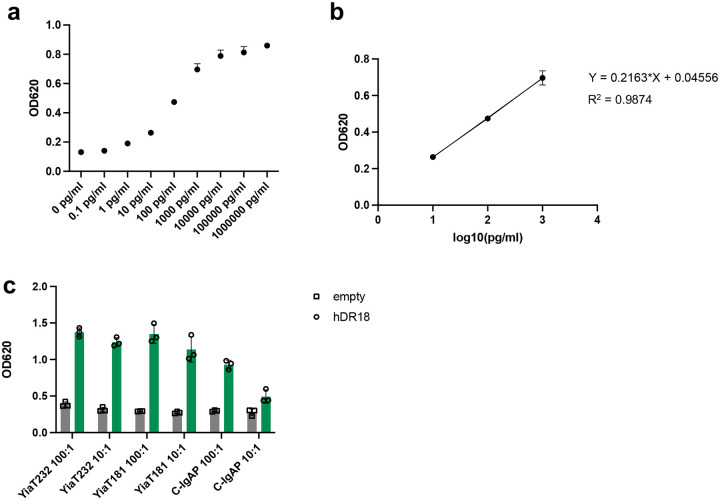
Clustering hDR18 on the surface of bacteria mediates enhanced activation of the IL18 receptor. **a**, The activity of purified hDR18 at different concentrations. **b**, Linear regression of purified hDR18 activity from 10pg/ml to 1000pg/ml. **c**, IL18 activity of bacteria control or bacteria displaying hDR18 by scaffold C-IgAP, YiaT232 or YiaT181 in MOI = 10 or MOI = 100. The activities of hDR18 were measured by HEK-Blue^™^ IL18 reporter cells.

**Fig. 4 |:**
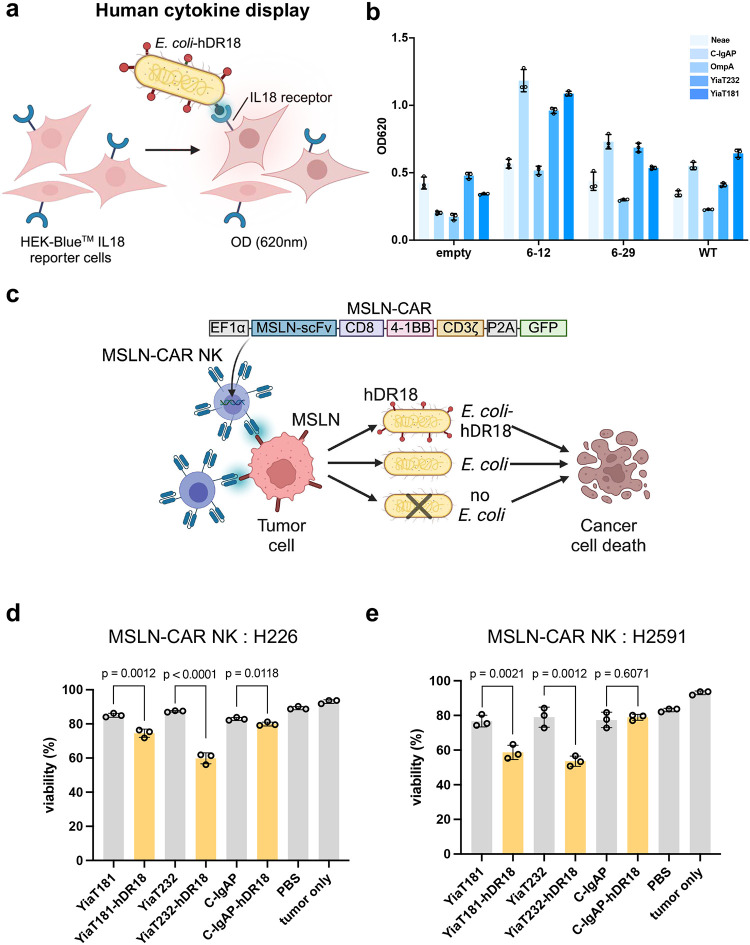
Bacteria displaying human decoy-resistant IL18 (hDR18) enhance anti-tumor responses of mesothelin (MSLN)-CAR NK cells. **a**, Schematic figure showing the use of HEK-Blue^™^ IL18 reporter cells to screen multiple bacterial scaffolds expressing human decoy-resistant IL-18 (hDR18) for activation. **b**, The activity (assessed using HEK-Blue^™^ in (**a**) of wildtype human IL18 (WT) and two variants of hDR18 (6–12 and 6–29) displayed by five bacterial scaffolds: Neae, C-IgAP, OmpA, YiaT232, YiaT181 with the multiplicity of infection (MOI) of *E. coli* displaying cytokines and HEK-Blue^™^ IL-18 reporter at 10. Human IL18 activity is represented by OD620. **c**, The schematic figure for *in vitro* coculture killing assay with MSLN-CAR NK and tumor cells. Briefly, MSLN-CAR NK cells were primed by bacteria or other control groups overnight and then cocultured with the tumor cells for 4 hours, viability of the tumor cells was assessed by Zombie NIR^™^ viability dye. **d**, **e**, Viability of H226 (**d**) and H2591 (**e**) cell lines after co-culture with MSLN-CAR NK primed by hDR18 displaying bacteria (YiaT232-IL18–6-12, YiaT232-hDR18; YiaT181-IL18–6-12, YiaT181-hDR18; C-IgAP-IL18–6-12, C-IgAP-hDR18) or control groups for overnight. The MOI of *E. coli* displaying cytokines and MSLN-CAR NK was 1000. E: T = 1:1. Unpaired t-test. Data are representative of three independent experiments with MSLN-CAR NK cells from three independent donors. Data represent means ± SEM (**d**, **e**).

To assess if this bacterial engineering approach would also work with human cytokines, we replaced the murine DR18 with a human decoy-resistant IL18 (hDR18) that was previously developed^25^. Based on our murine data, this could work both in combination with CAR T and NK cells, however, we chose CAR NK cells to avoid the possibility of exacerbating the risk of developing side effects like severe cytokine release commonly seen with CAR T cells^33–35^. We first evaluated the expression of wild type and two IL18 muteins (hIL18–6-12 and hIL18–6-29, both decoy resistant) in combination with different bacterial scaffolds using flow cytometry and assessed their activity upon co-culture with the HEK-Blue^™^ IL18 reporter cells (Fig. 4a). IL18–6-12 mutein (hereinafter referred to as hDR18) displayed by C-IgAP, YiaT232, or YiaT181 scaffolds had the highest IL18 activity (Fig. 4b). The optimized hDR18 displaying *E. coli* (YiaT232-hDR18 and YiaT181-hDR18) demonstrated high IL18 activity, with MOI of 10 equivalent to ~1500 pg/ml of soluble purified hDR18 (Extended Data Fig. 10a–c). Next, we assessed the ability of hDR18-displaying *E. coli* to enhance the cytotoxicity of the mesothelin targeting (MSLN) CAR NK cells generated from the peripheral blood of normal healthy volunteers using our optimized baboon lentivirus system^36^. We chose MSLN-CAR as proof-of-principle NK cell-based therapy as MSLN is highly expressed by several solid tumors including ovarian cancer, pancreatic cancer, and mesothelioma^37^. Activation of MSLN-CAR NK cells by engineered hDR18 bacteria (with different scaffolds) enhanced their cytotoxicity against MSLN^+^ cell lines (NCI-H226, and NCI-H259), bacteria displaying hDR18 with the YiaT232 scaffold were the most potent in enhancing the NK cell CAR cytotoxicity (p < 0.0001 towards H226, p = 0.0012 towards H2591) and were therefore chosen for further evaluation (Fig. 4c–e).

**Fig. 5 |:**
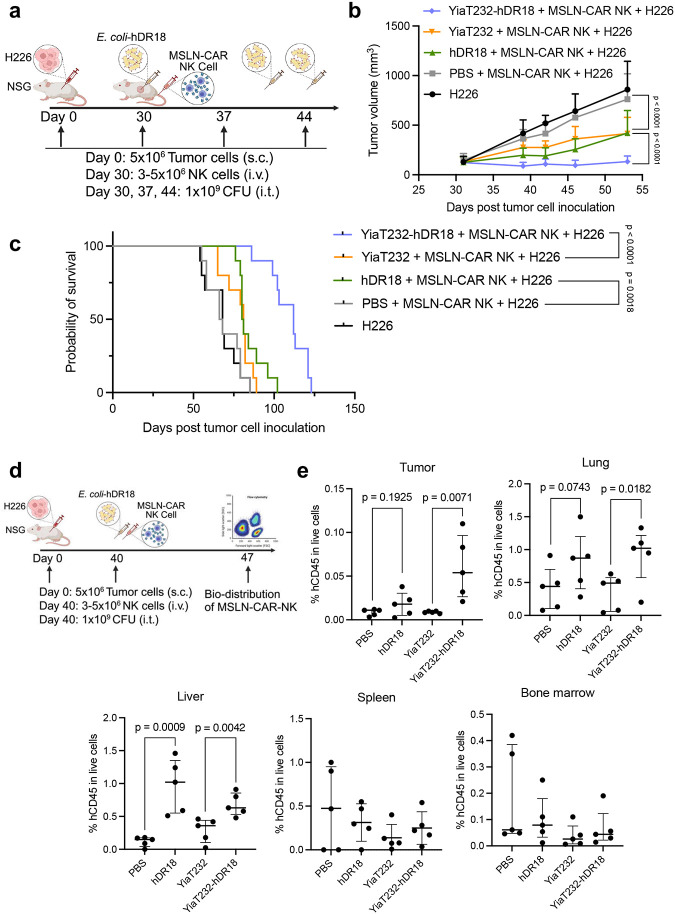
Bacteria displaying hDR18 enhance the proliferation, tumor trafficking, and efficacy of MSLN-CAR NK cells *in vivo* leading to improved tumor control. **a**, NOD scid gamma (NSG) mice were subcutaneously (s.c.) engrafted with 5×10^6^ H226 cells. Starting on day 30, mice (n = 10 each group) were treated with PBS, YiaT232 (10^9^ CFU), YiaT232-hDR18 (10^9^ CFU) and purified hDR18 (4mg/kg) (i.t.) three times (on days 30, 37 and, 44). 3–5 million MSLN-CAR NK cells were administrated intravenously (i.v.) except for mice in the tumor-only groups. All the mice were injected with 75 kU human recombinant IL2 every other day intraperitoneally (i.p.) to support the survival of human NK cells *in vivo*. **b, c**, Mean tumor growth (**b**) and Kaplan–Meier survival curves (**c**) for the tumor-bearing after treatment. Mean tumor growth and survival curves are the combination of two independent experiments, with n = 10 mice per group. Data represent means ± SEM. **d**, NSG mice were subcutaneously (s.c.) engrafted with 5×10^6^ H226 cells, on day 40, mice (n = 5 each group) were treated with PBS, YiaT232 (10^9^ CFU), YiaT232-hDR18 (10^9^ CFU) and purified hDR18 (4mg/kg) (i.t.). 5 million MSLN-CAR NK were administrated intravenously (i.v.) except in the mice from tumor only groups. All the mice were injected with 75kU human recombinant IL2 every other day intraperitoneally (i.p.). On Day 47, mice were sacrificed and organs (tumor, liver, lung, spleen, and bone marrow) of all mice were collected for analysis by flow cytometry. **e**, The percentage of human CD45^+^ cells in livers, tumors, spleens, lungs, and bone marrow post-treatment. Two-way ANOVA test for tumor growth curve (**b**) and Mantel-Cox test for survival curve (**c**), unpaired t-test for NK percentage (**e**).

Based on the above promising *in vitro* results, we hypothesized that the combination of the bacteria with MSLN-CAR NK cells will also lead to enhanced tumor control *in vivo* in a xenograft mouse model. Furthermore, based on the increased NK cell numbers that we saw in the tumors from immune-competent mice upon their treatment with the engineered bacteria (Fig. 3b), we also hypothesized this approach would help home adoptively transferred CAR NK cells into tumors and thus potentially overcome one of the major challenges in adoptive cell therapies, namely their poor trafficking particularly in the solid tumors. To assess this, we used nonobese diabetic scid gamma (NSG) mice bearing otherwise highly resistant NCI-H226 (mesothelioma) tumor cells subcutaneously and injected the engineered bacteria intratumorally (Fig. 5a). 5×10^6^ H226 cells were injected subcutaneously (flanks) and on day 30 post-tumor inoculation when the tumor size reached 80–200 mm^3^ the mice received 3–5×10^6^ CAR NK cells intravenously (tail vein injection). YiaT232-hDR18 (1×10^9^ CFU) or purified hDR18 (4mg/kg) versus PBS control were administrated intratumorally (i.t.) on days 30, 37 and 44 post-tumor inoculation. None of the mice had any apparent toxicity from the bacteria despite being highly immune compromised. Importantly, the treatment with YiaT232-hDR18 significantly enhanced tumor control (p<0.0001) and prolonged the survival (median survival = 112) in these mice outcompeting the cohort treated with the purified cytokine (hDR18, median survival = 80.5) (Fig. 5b, c).

To further investigate the biodistribution and tumor infiltration of MSLN-NK in these mice, a separate cohort of NSG mice was treated in the same fashion with similar tumors, and treatments (as in Fig. 5), except they received only one injection of the engineered bacteria, hDR18 or PBS control and then a week later sacrificed for further analyses (Fig. 5d). Treatment with YiaT232-hDR18 led to a significant increase in adoptively transferred MSLN-CAR NK cells into the tumors (p = 0.0071), with the purified hDR18 having only a limited impact (Fig. 5e). There was also an increase in the CAR NK cells in the liver and lungs with YiaT232-hDR18 bacteria and hDR18 suggesting this treatment could boost the proliferation of CAR NK cells *in vivo* (Fig. 5e).

These data support the assumption that *E. coli* expressing hDR18 YiaT232 scaffold (YiaT232-hDR18) are effective in enhancing anti-tumor responses of the CAR NK cells by their direct activation and increasing their tumor infiltration, thus making this combinatorial treatment approach attractive for potential translation in future clinical trials.

### CAR NK cell gene expression analysis

**Extended Data Fig. 11 |:**
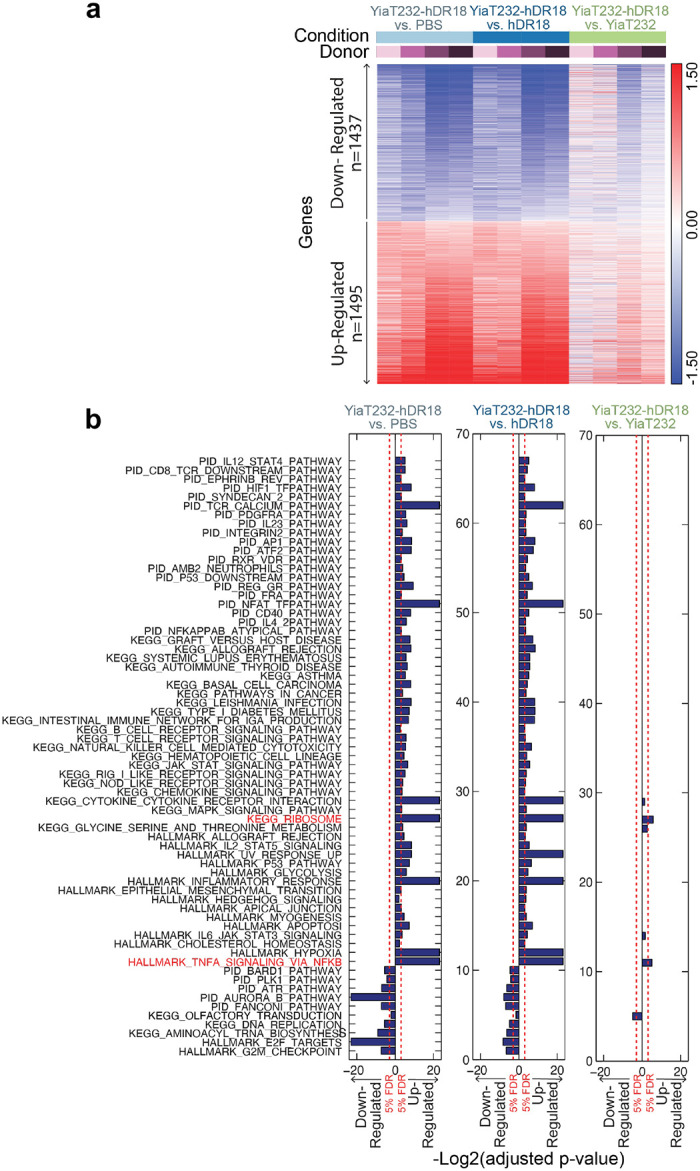
Gene expression changes in the MSLN-CAR NK cells induced by priming with *E. coli*-hDR18. **a**, Log2 fold changes for differentially expressed genes (YiaT232-hDR18, *E. coli* vs PBS, 5% FDR, n=2923). Fold changes were calculated for YiaT232-*E. coli*, YiaT232-hDR18, *E. coli* and hDR18 compared with PBS (paired). Data is row normalized. **b**, GSEA enrichment FDR values for the comparisons YiaT232-hDR18, *E. coli* vs PBS, YiaT232-*E. coli* and hDR18. The left panel represents down-regulated gene sets, and the right panel represents up-regulated gene sets. The dashed line depicts 5% FDR. Highlighted in red are gene sets that passed 5% FDR in all comparisons.

**Extended Data Fig. 12 |:**
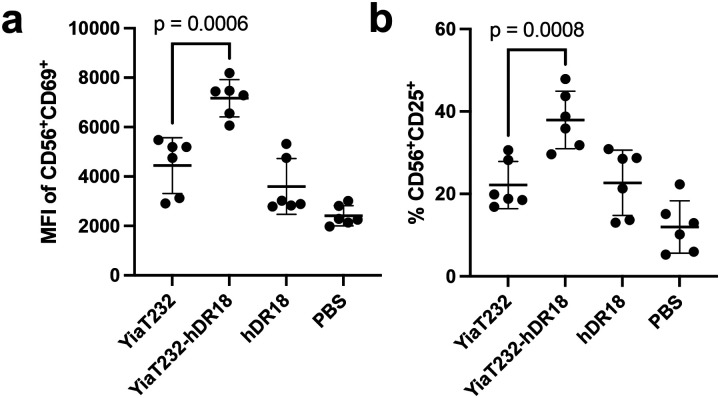
Bacteria displaying human decoy-resistant IL18 (hDR18) mediate activation of NK cells (PBMCs gated on NK cells) as assessed by expression of CD25 and CD60 on NK cells. **a**, The Median Fluorescence Intensity (MFI) of CD69^+^ cells in CD56^+^ cells. **b**, The percentage of CD25^+^ cells in CD56^+^ cells. Unpaired t-test. PBMCs were harvested from three donors. Data represent means ± SEM (**a**, **b**).

**Extended Data Fig. 13 |:**
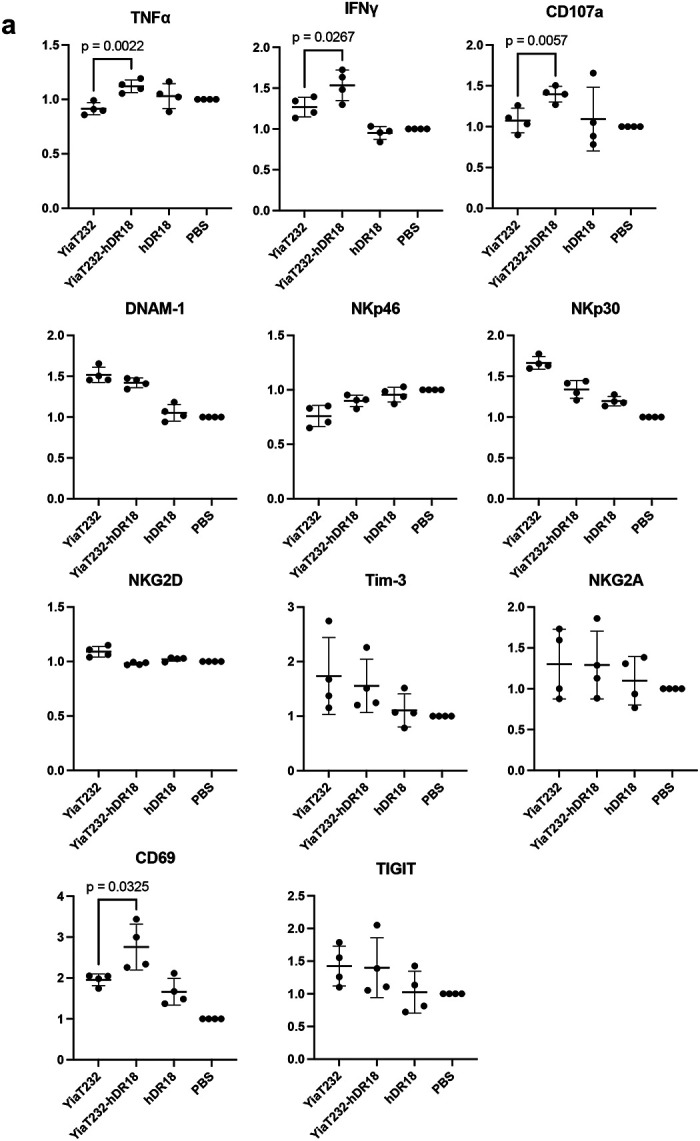
Bacteria displaying human decoy-resistant IL18 (hDR18) increase the expression of key activation markers in primary NK cells. **a**, The fold change of the percentage of TNFα^+^, IFNγ^+^, CD107a^+^, DNAM-1^+^, NKp46^+^, NKp30^+^, NKG2D^+^, Tim-3^+^, NKG2A^+^, CD69^+^ and TIGIT^+^ cells of different treatment groups compared to PBS treated groups. Unpaired t-test. NK cells were harvested from PBMCs of two donors. Data represent means ± SEM (**a**).

**Extended Data Fig. 14 |:**
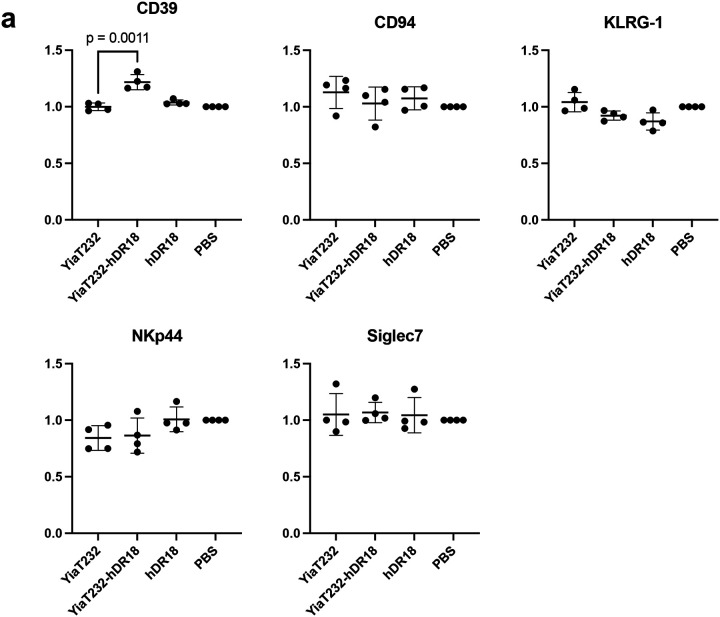
Bacteria displaying human decoy-resistant IL18 (hDR18) increase the expression of activation markers in primary NK cells. **a**, The fold change of the percentage of CD39^+^, CD94^+^, KLRG-1^+^, NKp44^+^ and Siglec-7^+^ cells of different treatment groups compared to PBS treated groups. Unpaired t-test. NK cells were harvested from PBMCs of two donors. Data represent means ± SEM (**a**).

**Fig. 6 |:**
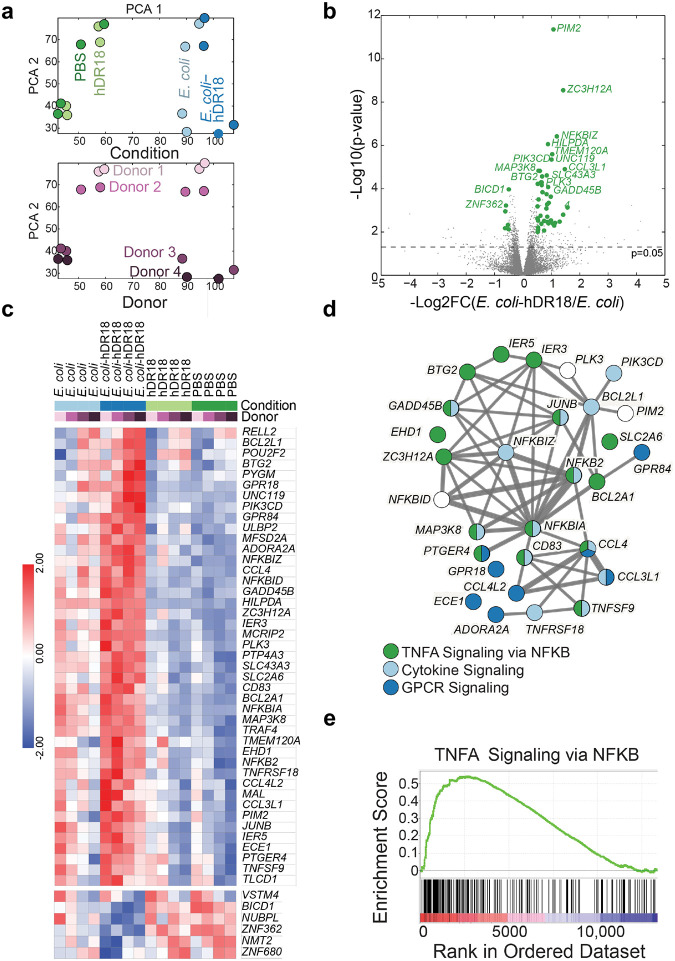
Gene expression changes in the MSLN-CAR NK cells induced by priming with *E. coli*-hDR18. mRNA transcript levels were measured in MSLN-CAR NK cells 73 hours after treatment with YiaT232-hDR18, *E. coli* (MOI = 1000), YiaT232, *E. coli* (MOI = 1000), hDR18 (no bacteria, 100ng/ml) and PBS (**a**, **b**, **c**, **d**, **e**). **a**, Principal component analysis of genes that were differentially expressed between YiaT232-hDR18, *E. coli,* and PBS (5% FDR, n=2923). Samples were labeled with the different treatment groups (Top) and different donors (Bottom). **b**, Volcano plot showing gene expression of YiaT232-hDR18, *E. coli,* and YiaT232-*E. coli* treated MSL-CAR NK cells. Highlighted in green are genes that passed 5% FDR comparing YiaT232-hDR18, *E. coli,* and PBS with log2FC>0.5 (or log2FC<−0.5) and had p-value<0.01 comparing YiaT232-hDR18, *E. coli and* YiaT232, *E. coli* with log2FC>0.5 (or log2FC<−0.5). n=43 up-regulated, n=6 down-regulated genes. The dashed line depicts a p-value of 0.05. **c**, Normalized expression levels (z-scores) of the genes highlighted in B. **d**, String network analysis for the upregulated genes highlighted in B (n=43). Genes were labeled according to the significantly enriched msigDB gene sets; *TNFα* signaling via *NF*κ*B*, cytokine signaling, and GPCR signaling. **e**, GSEA enrichment plot for the KEGG *TNFα* signaling via *NF*κ*B* in YiaT232-hDR18, *E. coli,* and YiaT232-*E. coli* groups.

To better understand potential mechanism(s) mediating enhanced CAR NK cell anti-tumor responses, we performed RNA sequencing (RNAseq) of MSLN-CAR NK cells, co-cultured with the engineered bacteria. MSLN-CAR NK cells were primed for 3 hours with bacteria expressing hDR18 (YiaT232-hDR18, MOI = 1000), bacteria expressing empty YiaT232 scaffold (YiaT232, MOI = 1000), soluble purified hDR18 protein (no bacteria, 100ng/ml), or PBS control. We focused our analysis on the group of differentially expressed genes, which passed 5% FDR (False Discovery Rate) in the YiaT232-hDR18 *bacteria* versus *PBS (*named *Y18-PBS) comparison groups (*Extended Data Fig. 11a). PCA (Principal Component Analysis), suggested that the first principle was dominated by the effect of the engineered bacteria on NK cells, and the second principle was dominated by the healthy donor variability (Fig. 6a). In the *Y18-PBS* group, we further studied the genes that were differentially expressed between YiaT232-hDR18 and YiaT232, (colored green in Fig. 6b and detailed in Fig. 6c). The interaction network for the upregulated genes is described in Fig. 6d, color-coded by the main gene sets that were found to have significant overlaps (MsigDB, investigate tool). These sets included ‘*TNFα* signaling via *NF*κ*B’* (green, padj=2.63e-23), ‘Cytokine signaling’ (light blue, padj=9.61e-6), and ‘*GPCR* signaling’ (dark blue, padj=2.48e-4). In addition, GSEA (Gene Set Enrichment Analysis) also identified ‘TNFα signaling via NFκB’ as one of the most significantly enriched pathways (adjp= 0.006, Fig. 6e and Extended Data Fig. 12b). To confirm this, we evaluated the activation of NK cells within PBMCs, primed for 12 hours using the above conditions, and then analyzed by flow cytometry. We found significant upregulation of the NK activation markers CD25 and CD69 (Extended Data Fig. 12a, b) as well as TNFα, IFNγ, and CD107a (Extended Data Fig. 13a, Extended Data Fig. 14a). With these data, we conclude that YiaT232-hDR18 bacteria can efficiently activate NK cells (with or without MSLN-CAR) leading to increased killing efficacy.

## Discussion

Here, we describe the development of a new immunotherapeutic platform using engineering non-pathogenic, tumor tropic, and pan-sensitive *E. coli* K-12 DH5α to surface display key activating cytokines and demonstrate the safety and promising efficacy of this approach in otherwise hard-to-treat tumors. *E. coli* K-12 DH5α displaying DR18 demonstrated tumor tropism, inducing dramatic CD8^+^ T and NK cell-dependent tumor controls, and curing a significant fraction of tumor bearing mice. The recall and abscopal effects suggest induction of immunologic memory and epitope spreading with this approach. The enhanced tumor trafficking and promising efficacy of the engineered bacteria when combined with systemically delivered CAR NK cells in NSG mice bearing subcutaneous tumors, demonstrate the potential of this approach to serve as a “tumor GPS” for the systemically delivered CAR treatments in solid malignancies. We also demonstrate the safety of the engineered bacteria in immune-competent and in combination with CAR NK cells in highly immune-deficient NSG mice. These findings highlight the feasibility, safety, and potential efficacy of this new immunotherapy platform, making a strong case for its evaluation in the clinic in patients with otherwise poor prognoses.

The major novelty of our study is the application of surface display of immune-activating cytokines on the bacterial outer membrane endowing this system to induce potent anti-tumor responses in contrast to the previous bacterial therapy studies using secretion and or self-lysis mediated delivery of biologics to the TME^12–15,17^. The surface display of biologics in live bacteria leads to significantly enhanced potency not only due to the tumor tropism of the bacteria but also its increased effective concentration due to the two versus three-dimensional mobility^38–40^ and prolonged half-life in the TME due to its continued biosynthesis by the live bacteria. *E. coli*. However, these studies have had limited efficacy in inducing strong and persistent immune responses, unless additional treatments like immune check point blockers were co-administered^30^. We used *E. coli* strain K-12. DH5α here because for several reasons; it is a facultative anaerobe which facilitates tumor tropism, it is pan-sensitive to multiple commonly used antibiotics, it lacks Horizontal Gene Transfer (HGT) machinery, and its genetic modification has included knocking out its recombinant system, which makes this strain harder to obtain any mutations for possible resistance to antibiotics^22,23,41–44^. Furthermore, it also lacks pks gene islands, present in E. *coli* Nissle 1917 and known to produce colibactin, a genotoxin associated with colorectal cancer^45^.

**Extended Data Fig. 15 |:**
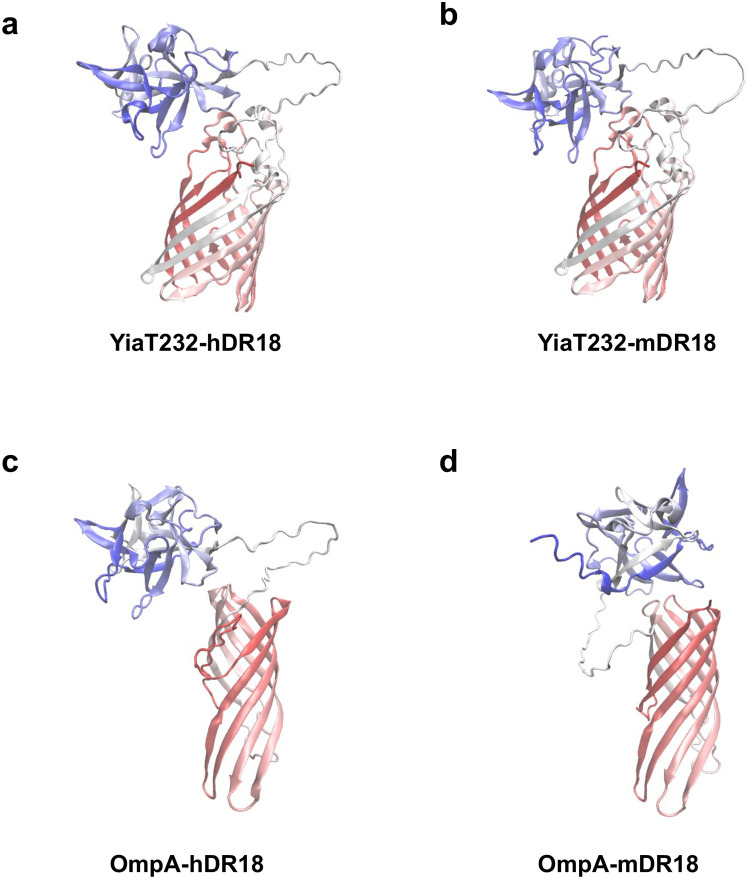
Protein Structure of **a**, YiaT232-hDR18. **b**, YiaT232-mDR18. **c**, OmpA-hDR18. **d**, OmpA-mDR18, predicted by Colabfold based on Alphafold 2.

A previous study has demonstrated superior efficacy of the soluble DR18 over regular IL18 in inducing anti-tumor repsonses^25^. We saw the bacteria displaying DR18 were not only better tolerated but also vastly superior to the soluble DR18, inducing a potent immune response leading to a cure in a significant proportion of tumor bearing animals. The surface displaying of this cytokine on the bacterial outer membrane may lead to its enhanced effective concentration and half-life in close proximity to the effector immune cells in TME along with the potential “adjuvant” function of the bacteria. The differential efficacy of bacterial scaffold proteins for murine versus human DR18 is most likely related to the steric restrictions of their spatial display affecting ligand-receptor interactions (Extended Data Fig. 15). Furthermore, the rechallenge experiments and abscopal effects suggested induction of immunological memory and epitope spreading leading to systemic immune responses and thus further increasing the application of our approach to patients with advanced metastatic tumors.

The immune profiling of the tumors after the bacterial therapy demonstrated the ability of these bacteria to significantly modulate otherwise highly immune suppressive TME, particularly in solid tumors like melanoma and colorectal carcinoma models. While we saw increased infiltration of granulocytes into the tumors, our depletion studies demonstrated the anti-tumor tumor responses to be mediated primarily by CD8^+^ T and NK cells, possibly along with a decrease in TAM frequency in the TME. Bacteria have been known to reduce TAMs through pyroptosis and TNFα-mediated killing of macrophages and thus contribute to their decreased frequency in the tumors observed after our bacterial therapy^46^. The increased homing of the adoptively transferred CAR NK cells and enhanced tumor control seen in xenograft mice bearing otherwise very resistant mesothelioma tumor cells with the bacteria displaying human DR18 further demonstrate the potential application of this approach in combination with CAR therapy. Our RNA-sequencing data suggest that the increased cytotoxicity of these cells is mainly regulated by *TNFα* signaling via *NFkB,* cytokine signaling, and *GPCR* signaling pathways. The *NFkB* pathway is well known for its crucial role in regulating the proliferation and activation of effector NK and T cells^47^. Indeed, examination of the core genes of the GSEA analysis for the pathway *TNFα* signaling via *NFkB*, identifies key activation genes such as *TNFSF9* (*41BB* ligand), *TNFα*, *CXCL10* (an inflammatory chemokine), *CD69* (activation signaling), *IL15RA* (receptor for IL-15), *TNFRSF9* (*41BB*), *STAT5A (*transcription factor), *IFNGR2* (*IFN*g receptor 2), *MYC* (transcription factor) and more. Similarly, CXCL10 is well known to induce NK cell trafficking into the tumors^48^ thus potentially explaining the increased trafficking of the CAR NK cells seen in our study.

While we saw the induction of potent immune responses that were associated with modulation of the key immune cells with the bacterial therapy, it was reassuring to see no major side effects and relatively low levels of cytokines including IL1γ, IL2, and IL6 known to mediate cytokine release syndrome^31,32,34,35^. Similarly, the bacteria did not cause an overwhelming systemic infection even in immune-compromised NSG mice supporting the potential safety of this approach even in patients with otherwise impaired immunity. The pan-sensitive nature of the *E. coli* K-12 DH5α to multiple commonly used antibiotics adds an additional layer of safety^23,44^.

In summary, we developed a novel live bacteria therapy-based immunotherapy platform using *E. coli* K-12 DH5α bacteria displaying key cytokines. The dramatic anti-tumor responses along with the safety seen with these bacteria in otherwise hard-to-treat tumors make this approach quite promising and build a strong case for their evaluation alone or in combination with other immunotherapeutic approaches including CAR T/NK cells and or immune-checkpoint blockade in the clinic.

## Methods and materials

### Cell culture

OVCAR8, K562 and Raji cells were purchased from American Type Culture Collection (ATCC; Rockville, MD). HEK-Blue^™^ IL18 cells were purchased from InvivoGen. H226 and H2591 were requested from Dr. David Barbie’s lab at Dana-Farber Cancer Institute (Boston, MA). MC38 and B16F10 were requested from Dr. Darrell Irvine’s lab at the Koch Institute (Cambridge, MA). HEK-Blue^™^ IL18 cells, MC38, and B16F10 were maintained in complete Dulbecco’s modified Eagle’s medium (DMEM; Corning, NY) supplemented with 10% fetal bovine serum (FBS; Corning, NY) and 100 U/ml penicillin-streptomycin (P/S; Corning, NY). For HEK-Blue^™^ IL18 cells, 100 μg/ml Normocin and 1X HEK Blue selection were added to maintain the cell lines. Raji, K562, OVCAR8, H226, and H2591 were cultured in Roswell Park Memorial Institute Medium-1640 (RPMI-1640; Corning, NY) supplemented with 10% FBS, 100 U/ml P/S, and 1x NEAA. All the cell lines mentioned were maintained at 37 °C in a humidified incubator with 5% carbon dioxide (CO_2_). Cells in passages 2–10 were used for the experiments.

### Escherichia coli surface display and protein structure prediction

For the surface display of cytokines (IL15, IL18, DR18, and IL21) we used the following plasmids: pLyGo-Ec-7 (Addgene#: 163135) with N-terminal fusion scaffold protein C-IgAP, pLyGo-Ec-8 (Addgene#: 163136) with C-terminal fusion scaffold protein Lpp-OmpA, pDSG323 (Addgene#: 115594) with C-terminal fusion scaffold protein Neae, and pDS861(a generous gift from Quintara Biosciences) with C-terminal fusion scaffold protein YiaT232 or YiaT181. DNA sequences for the encoding cytokines including 3 copies of GGGGS linker between scaffolds and the cytokines with DYKDDDDK-tag (FLAG-tag) in the N-terminal and Myc-tag in the C-terminal were inserted between two SapI sites for pLyGo-Ec-7 and pLyGo-Ec-8, between SpeI and PstI sites for pDSG323 and between NotI and BamHI sites for pDS861 by NEBuilder^®^ HiFI DNA Assembly Master Mix (NEB, catalog#: M5520AVIAL). For the optimization of the surface display of Lpp-OmpA-mDR18, the high-copy plasmid pDS861-dsRed (from Quintara Biosciences) was digested with XbaI and NotI. The *E. coli* rhaSR-PrhaBAD inducible promoter system containing regulator proteins (RhaR and RhaS), a rhamnose-responsive promoter (P_rhaBAD_), a ribosome binding site (RBS), along with Lpp-OmpA-mDR18 were amplified from pLyGo-Ec-8-mDR18. The DNA parts mentioned above were assembled with digested pDS861-dsRed backbone by NEBuilder^®^ HiFi DNA Assembly Master Mix. All plasmids were validated by Sanger sequencing before the next steps. The DNA fragments for cytokines with an N-terminal Myc tag and a C-terminal FLAG-tag were synthesized by Twist Bioscience (CA) or 3 copies of GGGGS linkers were introduced by primers.

For bacterial induction, *E. coli* K-12 DH5α with the corresponding plasmid were inoculated in fresh Luria-Bertani (LB) medium with 50 μg/ml Kanamycin. After overnight culture in a shaker (37 °C, 250 rpm), bacteria suspensions were diluted by 10-fold in the fresh LB with 50 μg/ml Kanamycin and 10 mM L-Rhamnose for derivatives of pLyGo-Ec-7, pLyGo-Ec-8 and pDS861 and in the fresh LB with 50 μg/ml Kanamycin and 100 ng/ml anhydrotetracycline (aTc) for modified plasmids based on pDSG323. After 48-hour induction in a shaker (25 °C, 250 rpm), bacteria were collected for the next step. For the optimized induction of hDR18 displayed by YiaT232, YiaT181, and C-IgAP, overnight bacterial culture was diluted ten times in the fresh LB containing 50 μg/ml Kanamycin and 10mM L-rhamnose monohydrate, and proteins were induced at 25 °C, 250 rpm for 72–96 h. Bacteria were collected after induction for the next step. For bacteria surface display verification, 20μl bacterial suspension was collected, washed once with PBS, and then incubated with anti-DYKDDDDK Tag Antibody (BioLegend, catalog# 637315) in FACS buffer (2% FBS, 0.1% w/v sodium azide, 2mM Ethylenediaminetetraacetic acid dissolved in DPBS) for 20 min at RT, washed 2x and then suspended in PBS for flow cytometry.

The structures of various fusion protein were predicted by ColabFold. The structure images was analyzed by Visual Molecular Dynamics (VMD)^49^.

### Protein expression and purification

Murine DR18, murine IL15, and human DR18, were cloned into pSH200 vector (a generous gift from Prof. Xiling Shen at Duke University) containing 6xHistidine tag (His-tag) via the Gibson assembly after digestion with BamHI and XbaI. Plasmids were verified by Sanger sequencing before expression. The cytokines were expressed and purified as previously described^50–52^. Briefly, verified plasmids were transformed into E. coli BL21 (DE3), which were cultured in 20 ml fresh LB with 100 μg/ml ampicillin at 37 °C, 250 rpm. After overnight growth, bacteria were added to a flask of 500ml fresh LB with 100 μg/ml ampicillin and grew in a shaker (37 °C, 200 rpm) to reach an OD600 of 0.6 ~ 0.8. Isopropyl ß-D-1-thiogalactopyranoside (IPTG) was added to LB until the final concentration was 1 mM and the whole flask was shaken in the condition of 20 °C and 200 rpm for 20h before the bacteria pellet was collected, washed 3 times with 1x PBS, resuspended in 15 ml binding buffer (3x PBS, 20 mM imidazole, pH=7.4) and stored in −80 °C before bacteria was lysed by lysis buffer (3x PBS, 20 mM imidazole, 20 mM PMSF, 1% v/v Triton X-100, 1% v/v EDTA-free Halt^™^ protease inhibitor cocktail (ThermoFisher, catalog #: 78430), 0.1% w/v lysozyme) and sonication subsequently to release produced protein. The produced protein was sequentially purified by affinity chromatography using Ni-NTA agarose beads (GOLDBIO, catalog #: H-350–25) with endotoxin removal step exerted by 0.1% Triton X-114 and fast protein liquid chromatography (FPLC, NGC Quest 10 Chromatography system, Biorad, Hercules, CA). Protein fractions detected at λ = 280 nm were collected. Protein concentrations in the fractions were quantified by detergent compatible (DC) protein assay (Bio-Rad Laboratories, Hercules, CA) according to the manufacturer’s instructions, and purities were verified by SDS-PAGE. Validated protein was aliquoted and kept at −80 °C in the buffer of 3x PBS with 10% glycerol and 1mM Dithiothreitol (Fisher, catalog #: BP172–5) until further use.

### Animal experiments

6–8-week-old female C57BL/6J (JAX stock no. 000664) mice and 4-week-old female NOD.Cg-Prkdc^scid^ Il2rg^tm1Wjl^/SzJ (NSG^™^, JAX stock no. 005557) were purchased and maintained in the animal facility at Northeastern University (NEU). All animal studies and procedures were performed following federal, state, and local guidelines under institutional animal care and approved by the Institutional Animal Care and Use Committees (IACUC) at NEU.

For scaffold screening experiment, 0.5 million MC38 in 30μl sterile PBS were injected subcutaneously into both the flanks of C57BL/6 mice. On day 7, when tumor volume reached 50–100 mm^3^, mice were injected intratumorally with PBS, purified protein, or engineered bacteria in 20μl sterile PBS twice weekly for a total of 3 injections into tumors on both flanks.

For the intratumoral injection experiments (Fig. 1c, Extended Data Fig. 5a), 0.5 million MC38 or B16F10 in 30μl sterile PBS were injected into the subcutaneous flank of C57BL/6 mice. On day 7, when tumors reached 40–70 mm^3^ for B16F10 and 50–100 mm^3^ for MC38, mice were injected intratumorally with PBS, purified protein, or engineered bacteria in 20μl sterile PBS twice weekly for a total of 3 injections in MC38 and 5 injections in B16F10.

For the abscopal effect experiments (Fig. 2a), 0.5 million MC38 and 0.3 million MC38 in 30μl sterile PBS were injected subcutaneously into both the flanks of C57BL/6 mice (0.5 million on the left flank and 0.3 million on the right flank). On day 7, when tumors on the left flank reached 50–100 mm^3^, mice were injected intratumorally with PBS or engineered bacteria in 20μl sterile PBS twice weekly for a total of 5 shots.

For the systemic delivery experiments (Fig. 2d), one million MC38 in 30μl sterile PBS were injected subcutaneously into the flanks of C57BL/6 mice. On day 8, when the flank tumors reached 120–200 mm^3^, mice were injected intravenously with PBS or engineered bacteria in 100μl sterile PBS two times weekly for 4 shots in total.

For the *in vivo* experiments involving MSLN-CAR NK, five million H226 in 50μl sterile PBS were injected subcutaneously into the flanks of NSG mice. On day 30, when the flank tumors reached 80–200 mm^3^, 3 – 5 million MSLN-CAR NK cells (in 100μl sterile PBS) generated from 3 normal healthy donors were injected intravenously (tail vein). PBS, engineered bacteria, and purified protein (hDR18) in 20μl sterile PBS were injected intratumorally every week for 3 shots in total. To support human CAR NK cells, the mice received 75kU of human recombinant IL2 (Miltenyi Biotec, USA) intraperitoneally every other day. Tumor volume was measured by calipers and calculated by 0.5 x length x width^2^ for 2 – 3 times a week.

### Staining and FACS analysis

Antibodies against mouse Ly6c (clone HK1.4), CD11b (clone M1/70), F4/80 (clone BM8), CD45 (clone 30-F11), CD3 (clone 17A2), CD4 (clone GK1.5), CD8 (clone 53–6.7), NK1.1 (clone PK136), CD45.2 (clone 104), Ly6g (clone 1A8) and antibodies against human CD56 (clone HCD56), CD16 (clone 3G8), NKp30 (clone P30–15), DNAM1 (clone 11A8), NKp46 (clone 9E2), KLRG1 (clone 2F1), Siglec7 (clone S7.7), NKp44 (clone P44–8), CD94 (clone DX22), CD39 (clone A1), TIGIT (clone A15153G), Tim3 (clone F38–2E2), CD69 (clone FN50), NKG2A (clone S19004C), CD3 (clone OKT3), CD4 (clone RPA-T4), CD8 (clone SK1), CD25 (clone M-A251), Zombie NIR Fixable Viability Dye (cat. No. 423105) were purchased from BioLegend. Antibodies against human CD45 (clone HI30) were purchased from BD biosciences. All antibodies were diluted 1:100 – 1:50. The live/dead dye Aqua (cat. No. L34966), live/dead dye Violet (cat. No. L34955), live/dead dye Green (cat. No. L23101) were purchased from Thermo Fisher Scientific (diluted 1:1000 – 1:300).

**Extended Data Fig. 16 |:**
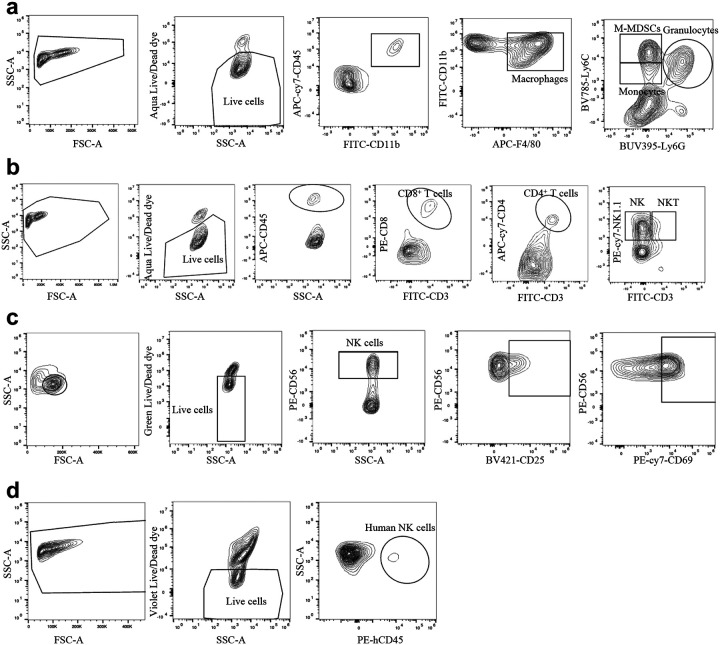
Flow cytometry gating strategy. **a**, **b**, The gating strategy for analyzing granulocytes, M-MDSCs, monocytes, macrophages (**a**), CD4 T cells, CD8 T cells, NK cells, and NKT cells (**b**). The cells were harvested from tumors of mice bearing MC38 cells. **c**, The gating strategy for analyzing NK cells in human PBMCs. d, The gating strategy for analyzing MSLN-CAR NK cells in NSG mice.

Tumor-bearing mice were euthanized and tumors, lungs and livers sliced and digested by digestion buffer (RPMI-10 with 5% FBS, 10mM HEPES, 100μg/ml penicillin-streptomycin, 20μg/ml DNase I and 1mg/ml collagenase IV) for 1h at 37 °C with agitation, followed by treatment with ammonium-chloride-potassium (ACK) buffer for red blood cell (RBD) lysis and then filtered through a 70μm strainer to remove debris. Spleen and bone marrow from rear limbs were mechanically dissociated and then filtered through a 70μm strainer and lysed by ACK buffer. Single cell suspension filtered through 70μm strainers without RBD were washed with PBS for 3 times, blocked by Fc blocker (BD biosciences, clone 2.4G2) at 4 °C for 10 minutes, washed with PBS for 3 times, and stained with live/dead dyes in PBS for 20 minutes at room temperature (RT) in the dark, washed with FACS buffer (PBS, 5mM Ethylenediaminetetraacetic acid, EDTA, 2% FBS, 0.1% NaN_3_ sodium azide) 3 times, and then stained with antibody mix for 30 minutes at RT in the dark, washed with PBS 3 times, fixed with 4% paraformaldehyde (PFA) in PBS for 15 minutes at RT in the dark, washed with PBS 3 times before running on a flow cytometer. Stained samples were analyzed by Sony ID7000 and FlowJo software (Flowjo LLC). Gating strategies were presented as shown in Extended Fig. 16.

### Luminex analysis

Blood samples were collected from the submandibular vein at two time points (3 days and 7 days) using BD Vacutainer blood collection tubes with lithium heparin. Heparinized plasma was obtained by centrifugation at 3,000 rpm for 15 minutes at 4 °C and sent to Eve Technology for Luminex analysis for measurement of cytokines and chemokines.

### In vivo cell depletions

Antibodies against CD8α (clone 2.43, BioXCell, 8mg/kg i.p. twice weekly), NK1.1 (clone PK136, BioXCells, 8mg/kg i.p. twice weekly), and Ly6g (clone 1A8, BioXCells, 8mg/kg i.p twice weekly) were used to deplete CD8^+^ T cells, NK cells and granulocytes respectively *in vivo*. Blood samples were collected from the submandibular vein, harvested in EDTA-coated tubes (BD biosciences), and lysed using LCK buffer to remove RBD. The cells were then stained and analyzed by flow cytometer as previously described for verification of cell type removal^53^.

### NK isolation and transduction

**Extended Data Fig. 17 |:**
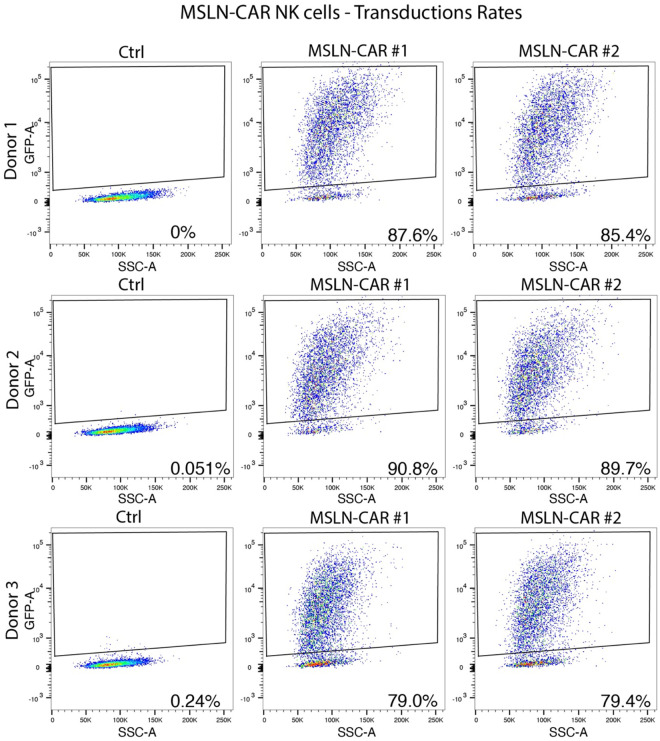
Transduction rates of MSLN-CAR NK cells

NK cells were isolated from healthy donor leukapheresis collars (Crimson core, ID T0197) via Ficoll-Paque density gradient using RosetteSep human NK cell enrichment kit (Stemcell technologies). Purified NK cells were cultured in NK MACS medium (Miltenyi Biotech) supplemented with 5% human serum and 500U/ml human IL2 (Miltenyi Biotech) and maintained at 37 °C in a humidified incubator with 5% carbon dioxide (CO_2_). MSLN-CAR gene construct was designed with the following components: signal peptide (CD8), MSLN scFv derived from the YP218 antibody clone^54^, transmembrane domains (CD8), followed by intracellular signaling domains, 4–1BB and CD3ξ. The CAR construct was linked to eGFP by P2A self-cleaving peptide, and it allowed the assessment of transduction efficiency. Lentiviral supernatants were produced using our baboon lentiviral (BaLV) system as reported previously^36^. 48hr post transduction, MSLN-CAR NK cells were stained for violet live/dead stain, and the transduction rates were evaluated by flow cytometry, gating on live, GFP^+^ cells (Extended Data Fig. 17).

### In vitro coculture assays

For HEK-Blue^™^ IL-18 cells, 54000 cells and bacteria with different MOI were added to each well of 96-well plate in 200μl DMEM supplemented with 10% FBS and 100U/ml P/S. After 20h incubation, 20μl media from each well was mixed with 180 μl Quanti-Blue for 10 – 20 minutes at 37°C, and optical density in 620nm was measured by a plate reader.

For NK cell killing assays, 15000 – 50000 effector NK cells (MSLN-CAR NK) labeled with Carboxyfluorescein succinimidyl ester (CFSE) dye were cocultured with bacteria with MOI of 1000 overnight (12–16h), and then cocultured with 50000 target tumor cells (H226, Raji, OVCAR8, K562 or H2591) for 4–6 hours. Afterwards, the cells treated with a detaching buffer (PBS, 2mM EDTA) and harvested for staining. Briefly, cells washed with PBS three times were stained with Zombie NIR Fixable Viability Dye for 15 minutes at RT in the dark. Afterwards, samples were washed three times with the FACS buffer before running on a flow cytometer.

For marker analysis, human PBMC were treated with the bacteria at MOI of 1000 for 12h and then collected for antibody staining. Briefly, samples were stained with live/dead dye Green and subsequently stained with the antibody cocktails with PBS or FACS washes in between. For the NK cell staining, 50000 primary human NK were treated by bacteria with MOI of 1000 for 12h and cocultured with 50000 target H226 for 6 hours. GolgiPlug and GolgiStop (Brefeldin A and Monensin, BD biosciences) were added 1 hour after the starting point of co-culture. After 6-hour co-culture, cells were collected, stained with live/dead dye Violet, and then stained with antibodies against surface markers before fixation with three washes of PBS or FACS buffer in between. Afterwards, the cells were fixed with BD Cytofix/Cytoperm^™^ Buffer (BD biosciences) for 30 minutes on ice, stained with intracellular antibodies in BD Perm/Wash^™^ Buffer (BD biosciences) for 30 minutes on ice with three washes of BD Perm/Wash^™^ Buffer or FACS buffer in between. Cells were acquired using Sony ID7000.

### RNA sequencing and analysis

1 – 3 million MSLN-CAR NK cells from four different donors (two donor per biological replicate) were cocultured with bacteria YiaT232 (MOI 1000), bacteria YiaT232-hDR18 (MOI 1000), purified hDR18 (100ng/ml) or PBS for 3h, after which cells were harvested for RNA extraction and RNA sequencing. The FASTQ files were processed using STAR alignment^55^ followed by DESeq2^56^. P-values and adjusted p-values were determined by DESeq2, and pathway analysis was performed using gene set enrichment analysis (GSEA^57,58^) for pre ranked lists (based on fold changes) and MsigDB^57,58^ focusing on genes with adjusted p-value<=0.05 in the comparison of NK cells cultured with YiaT232-hDR18 *E. coli* vs. PBS, with p-value<0.01 and log2FC>0.5 (or log2FC<−0.5) in the comparison of NK cells cultured with YiaT232-hDR18 *E. coli vs*. YiaT232.

### Statistics and reproducibility

Data were analyzed by Student’s t-test (two-sided), one-way, two-way ANOVA, Kaplan-Meier methods, or log-rank test by GraphPad PRISM. No statistical methods were applied to determine sample size. Mice were randomized into different groups after tumor inoculation. Mice with signs of sickness (hunched back, severe ulceration and more than 15% body weight loss) were immediately euthanized and excluded from the analysis. The investigators were not blinded to allocation during experiments and outcome assessments. RNAseq data was evaluated using p-values and adjusted p-values produced by DESeq2.

## Extended Data

## Figures and Tables

**Summary Fig. |:**
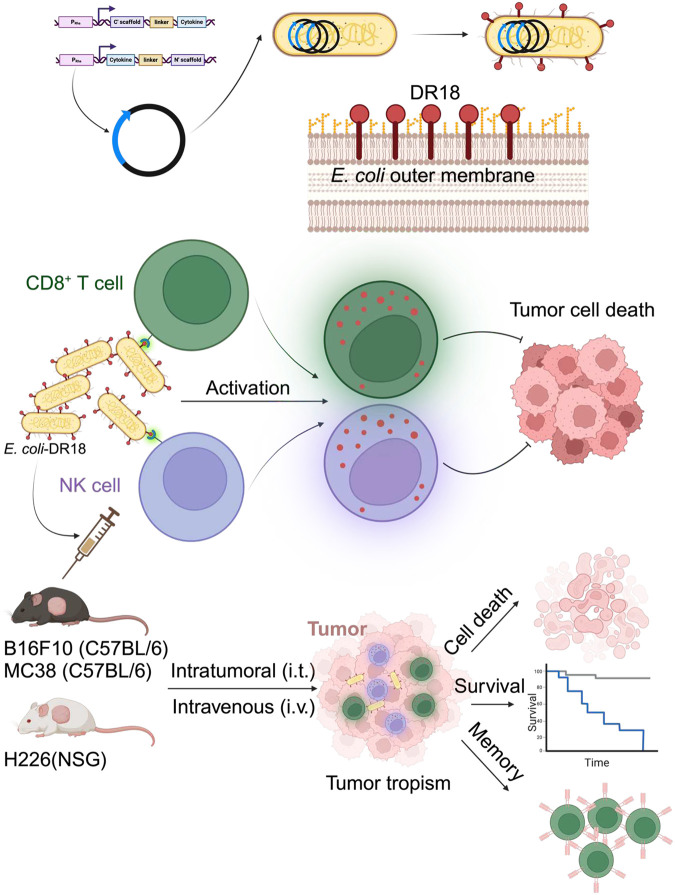
Schematic representation of our approach to surface display murine and human cytokines in non-pathogenic *E. coli* as a promising platform for immunotherapy, with *E. coli* displaying decoy-resistant IL18 mutein (DR18) being most effective *in vitro* and in immune-competent MC38 and B16 models and NSG mice bearing mesothelioma tumor cells treated with CAR NK cells.

## Data Availability

All data generated during this study are available within the paper. The bulk RNA-seq data will be deposited on Gene Expression Omnibus. Source data are provided with this paper.
